# Rac1-PAK1 regulation of Rab11 cycling promotes junction destabilization

**DOI:** 10.1083/jcb.202002114

**Published:** 2021-04-29

**Authors:** Jennifer C. Erasmus, Kasia Smolarczyk, Helena Brezovjakova, Noor F. Mohd-Naim, Encarnación Lozano, Karl Matter, Vania M.M. Braga

**Affiliations:** 1 National Heart and Lung Institute, Faculty of Medicine, Imperial College London, London, UK; 2 Institute of Ophthalmology, University College London, London, UK

## Abstract

Rac1 GTPase is hyperactivated in tumors and contributes to malignancy. Rac1 disruption of junctions requires its effector PAK1, but the precise mechanisms are unknown. Here, we show that E-cadherin is internalized via micropinocytosis in a PAK1–dependent manner without catenin dissociation and degradation. In addition to internalization, PAK1 regulates E-cadherin transport by fine-tuning Rab small GTPase function. PAK1 phosphorylates a core Rab regulator, RabGDIβ, but not RabGDIα. Phosphorylated RabGDIβ preferentially associates with Rab5 and Rab11, which is predicted to promote Rab retrieval from membranes. Consistent with this hypothesis, Rab11 is activated by Rac1, and inhibition of Rab11 function partially rescues E-cadherin destabilization. Thus, Rac1 activation reduces surface cadherin levels as a net result of higher bulk flow of membrane uptake that counteracts Rab11-dependent E-cadherin delivery to junctions (recycling and/or exocytosis). This unique small GTPase crosstalk has an impact on Rac1 and PAK1 regulation of membrane remodeling during epithelial dedifferentiation, adhesion, and motility.

## Teaser

Rac1 promotes malignancy. Oncogenic Rac1 and its effector PAK1 disrupt junctions via internalization of cadherin adhesion receptors by bulk fluid uptake. PAK1 phosphorylates a key regulator of intracellular trafficking, RabGDIβ, thereby modulating its affinity with specific partners (i.e., Rab11) to re-route cadherin away from cell–cell contacts.

## Introduction

The small GTPase Rac1 plays a key role in the regulation of cell–cell adhesion and epithelial function in health and disease. Rac1 is essential for the formation and maintenance of cadherin contacts and differentiated epithelial tissues (McCormack et al., 2013). Yet, in a cancer context, uncontrolled Rac1 activation often correlates with metastatic behavior and poor prognosis, with cell–cell contact disruption, cell detachment, and enhanced migration (Porter et al., 2016). In addition to upregulation of Rac1 protein and mRNA levels, dysfunctional Rac1 signaling in tumors is also achieved by point mutations that increase Rac1 activation and hyperactivation of endogenous Rac1 by upstream regulators (exchange factors, oncogenes, or growth factor receptors; Maldonado et al., 2020; Olson, 2018; Porter et al., 2016). The impact and relevance of Rac1 in tumor progression is consistent with the breadth of its various activating mechanisms and the variety of tumor types affected (Maldonado et al., 2020).

Here, we investigate the mechanisms by which inappropriate Rac1 activation perturbs cell–cell contacts as part of a malignancy program. In SCCf12 cells, activated Rac1 promotes E-cadherin internalization in a clathrin-independent manner (Akhtar and Hotchin, 2001). In normal keratinocytes, overexpression of active Rac1 requires signaling from its effector, PAK1, to remove E-cadherin from junctions (Lozano et al., 2008). PAK1 belongs to a family of serine/threonine kinases that has fundamental roles in different cellular processes (Kumar et al., 2017), including epithelial differentiation and morphogenesis in numerous organisms (Bahri et al., 2010; Pirraglia et al., 2010; Tay et al., 2010; Vlachos et al., 2015). Destabilization of cadherin-dependent junctions by PAK1 activation is consistent with the role of other PAK family members in the adhesion of tumor cell lines (Fram et al., 2014; Ismail et al., 2017; Morse et al., 2016; Selamat et al., 2015) and the well-established PAK1 function in promoting tumor migration and metastasis (Kumar and Li, 2016).

The cellular processes by which PAK1 activity could mediate junction disassembly are not known. Rac1/PAK1 signaling can activate ROCK1 and thus cell contraction, which could contribute to junction perturbation; however, our previous work shows that cells flatten out upon Rac1 expression, and inhibition of ROCK does not rescue Rac1-dependent defects (Lozano et al., 2008). We hypothesize two alternative mechanisms. First, PAK1 could phosphorylate proteins found at cadherin complexes and modulate their binding affinity and/or internalization, thereby weakening cell–cell adhesion. E-cadherin cytoplasmic tail has distinct motifs required for its internalization that are masked by the interaction with p120^CTN^ or β-catenin (Kowalczyk and Nanes, 2012). It is feasible that PAK1 phosphorylation of cadherin or catenins could destabilize the complex and facilitate E-cadherin internalization. Indeed, unique Ser/Thr phosphorylation sites on the E-cadherin cytoplasmic tail have been shown to enhance (Lickert et al., 2000; McEwen et al., 2014) or weaken (Dupre-Crochet et al., 2007) its interaction with β-catenin. Furthermore, binding between α-catenin and β-catenin is strongly reduced by casein kinase II phosphorylation of α-catenin (Escobar et al., 2015; Ji et al., 2009) or at different residues in β-catenin (Bek and Kemler, 2002).

Second, Rac1 and subsequent PAK1 activation could modulate the trafficking of E-cadherin complexes, per se. Different oncogenes and destabilizing stimuli are known to modify the turnover rate of E-cadherin complexes by accelerating their internalization or preventing recycling back to the cell surface (Goldenring, 2013; Kowalczyk and Nanes, 2012). The various routes by which E-cadherin can traffic to and from cell–cell contacts are controlled by Rabs, a family of small GTPases that coordinate the formation of intracellular vesicles and vesicular docking, fusion, and motility (Wandinger-Ness and Zerial, 2014). Rac1 engagement with trafficking machinery and Rab GTPase signaling could play a role in the destabilization of cadherin adhesion. Rac1 signaling is known to crosstalk with Rab GTPases via modulation of the localization and activity levels of each other (Bouchet et al., 2016; Chen et al., 2014; Diaz et al., 2014; Margiotta et al., 2017; Mori et al., 2014; Shim et al., 2010) or via shared activators or effectors (Bouchet et al., 2018; Carroll et al., 2013; Kunita et al., 2007; Topp et al., 2004).

We favor the possibility that Rac1 coordination with Rab upstream regulators may control Rab activation/inactivation cycling, which is strictly coupled to Rab localization at different vesicular compartments. Similar to Rho GTPases, Rabs are activated by guanine nucleotide exchange factors (GEFs), inactivated by GTPase-activating proteins (GAPs), and sequestered by Rab GTP-dissociation inhibitor (RabGDI; Stenmark, 2009). The brain-specific RabGDIα and the ubiquitously expressed RabGDIβ (Nishimura et al., 1994) retrieve an inactive Rab from the donor vesicular compartment and keep it in a cytosolic pool until its delivery to an acceptor vesicle or organelle, enabling localized Rab activation by GEFs (Shinde and Maddika, 2018).

We identify novel mechanisms by which Rac1 and PAK1 signaling disrupt E-cadherin adhesion in normal keratinocytes. Rac1 activation induces E-cadherin internalization via micropinocytosis in a PAK1-dependent manner: There is no dissociation of catenins, and cadherin complexes are not targeted for degradation within the timeframe analyzed. Our data indicate that regulation of E-cadherin trafficking occurs by two mechanisms. First, activation of Rab11 by Rac1. Rab11 operates at the crossroads between endocytic and exocytic transport: (i) slow recycling of internalized cargo to polarized regions of epithelial membrane and (ii) delivery of transmembrane proteins from the trans-Golgi network via exocytosis, which may or may not occur via the recycling compartment (McDermott and Kim, 2015; Welz et al., 2014). Second, a specific phosphorylation of RabGDIβ, but not RabGDIα, by PAK1. Such post-translation modification increases RabGDIβ affinity to selected Rabs, thereby interfering with specific trafficking routes. Thus, our data reveal novel PAK1 functions in intracellular trafficking with impact on the modulation of cell–cell contact in pathological conditions. In addition, the direct interplay between PAK1, RabGDIβ, and Rab11 has significant importance for other PAK1 functions that require membrane remodeling during motility, ruffling, and fluid uptake.

## Results

**Figure 1.**
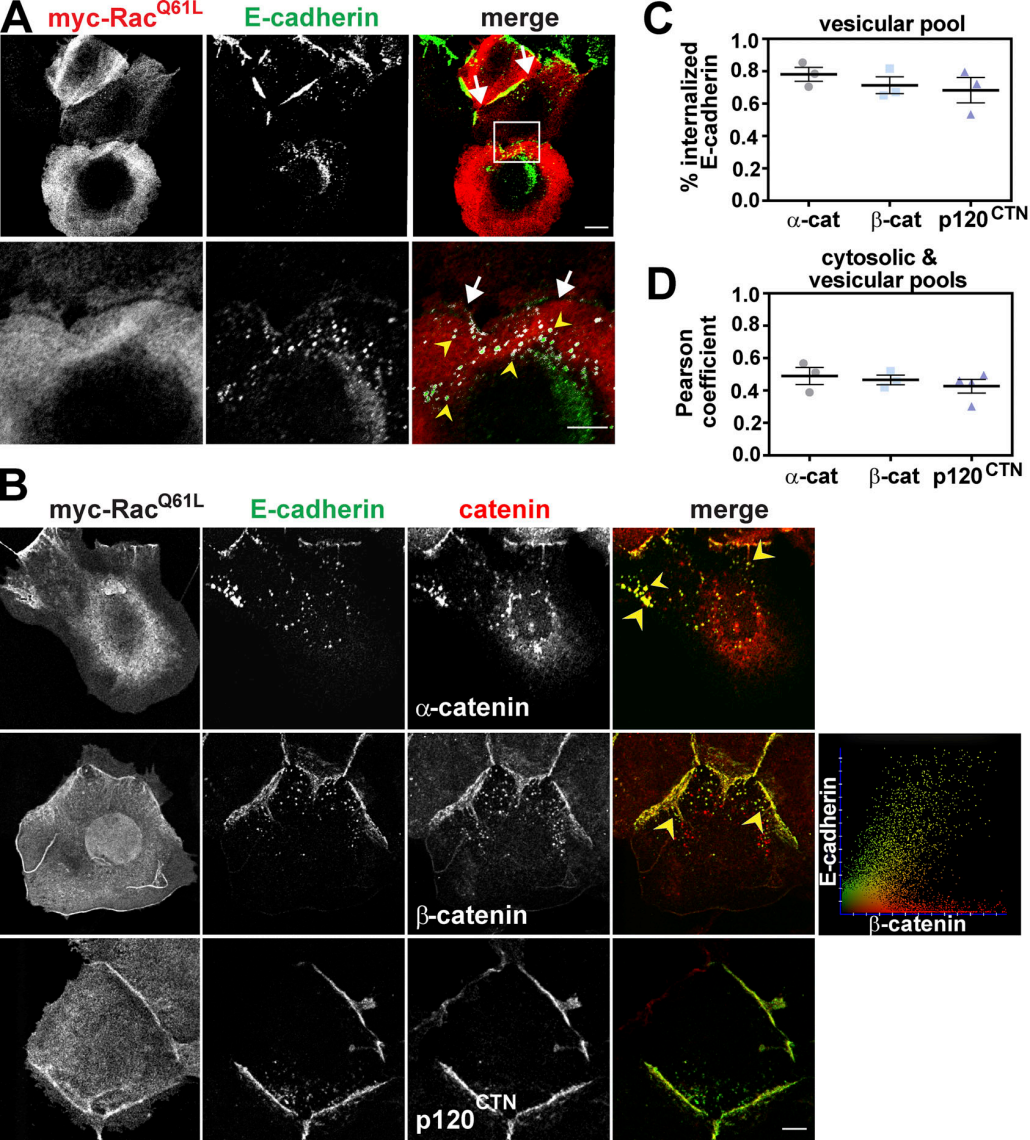
**Active Rac1 is internalized with E-cadherin and catenins.** Active Rac1 (pRK5-myc-Rac^Q61L^) was microinjected and expressed for 3 h. **(A and B)** Keratinocytes were fixed and stained with myc-tag and E-cadherin antibodies (A) or with antibodies against catenins (B). **(A)** E-cadherin and Rac^Q61L^ are co-internalized. **(B–D)** Colocalization of internalized E-cadherin and catenins. **(B)** Cells were labeled for active Rac1, cadherin and α-catenin, β-catenin, or p120^CTN^. **(C and D)** Quantification of internalized pools (see Materials and methods). **(C)** The percentage of E-cadherin pixels on intracellular vesicles that colocalize with catenins was quantified. **(D)** Pearson coefficient shows the colocalization of the internal pool of catenins (cytosolic and vesicular) with internalized E-cadherin (vesicular pool). Merged images are shown on the right columns (A and B) and zoom images are shown in the bottom row (A). Arrows show loss of E-cadherin at junctions, arrowheads point to vesicles containing E-cadherin and catenins. Scale bars = 2 µm. Images are representative of three independent biological experiments (thereafter *n* = 3), and error bars represent SD.

Keratinocytes expressing activated Rac1—constitutively active Q61L mutation, similar to the activating Q61L and G12V mutations found in oncogenic Ras—had junctions disrupted in a characteristic pattern: E-cadherin receptors were removed from the cell corners first (Fig. 1 A, arrows; Braga et al., 2000). Following activation of Rac1 in SCCf12 keratinocytes, enlarged vesicles containing E-cadherin complexes were observed (Akhtar and Hotchin, 2001). In our hands, normal keratinocytes also had numerous smaller intracellular vesicles in which E-cadherin and Rac1 colocalized (Fig. 1 A, yellow arrowheads). To understand the mechanism by which Rac1 promotes E-cadherin internalization, we initially assessed whether catenins were selectively released from internalized cadherin complexes. Normal keratinocytes were injected with constitutively active Rac1 (myc-Rac1^Q61L^) and costained for E-cadherin and catenins (Fig. 1 B). Images were segmented to eliminate junctional staining, and fluorescence signals in the resulting cytoplasmic area were quantified.

**Figure S1.**
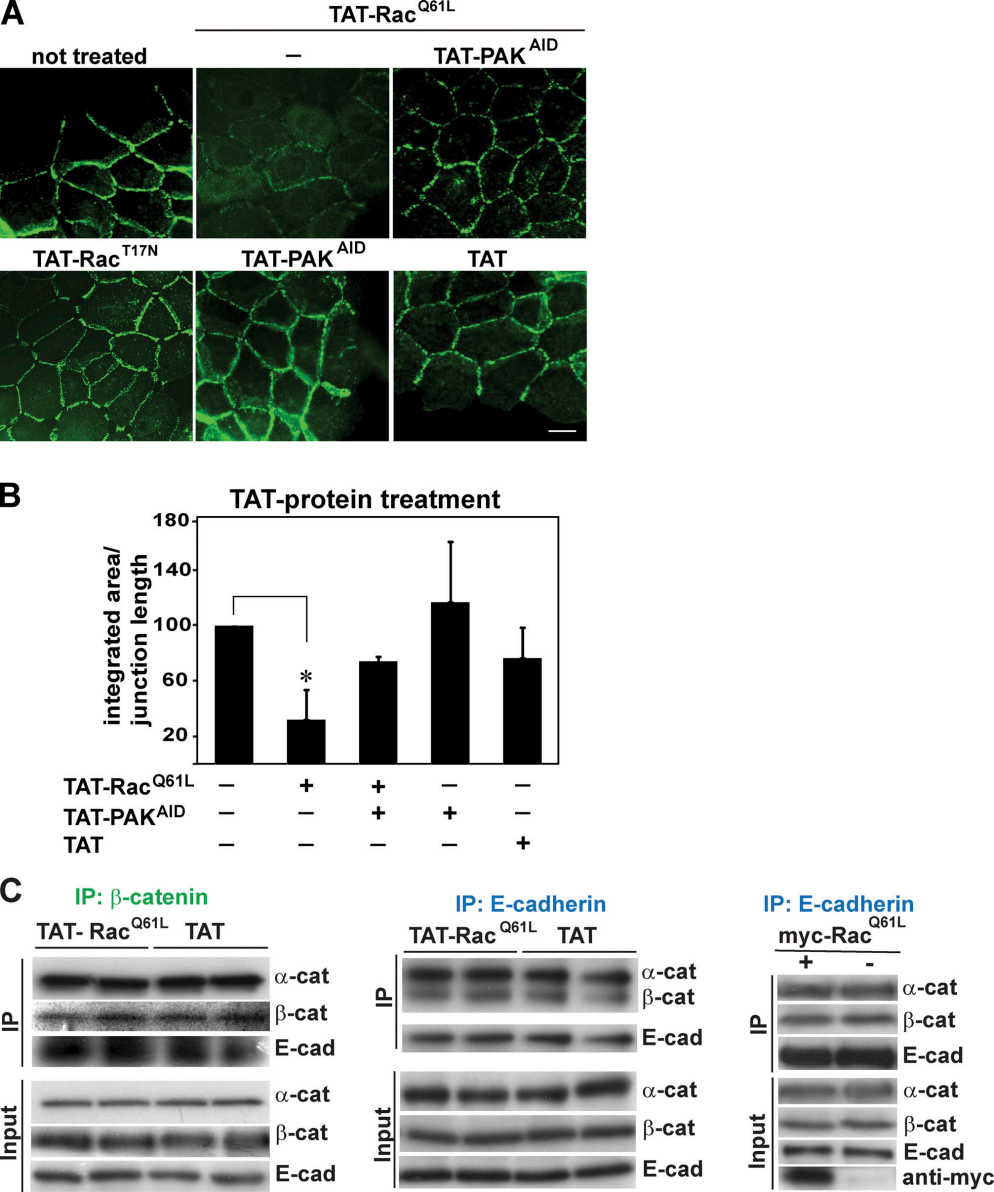
**Cellular effects of TAT proteins used in this study.** **(A)** Keratinocytes were left untreated or incubated with constitutively active Rac1 (TAT-Rac1^Q61L^) in the presence or absence of PAK1 autoinhibitory domain (TAT-PAK^AID^). Additionally, cells were treated with dominant-negative Rac1 (TAT-Rac1^T17N^), TAT-PAK^AID^, or TAT peptide by themselves. Cells were fixed and stained for E-cadherin. **(B)** Quantification of the phenotypes shown in A. Intensity levels of E-cadherin at cell–cell contacts were measured and normalized to the length of each junctions. Nontreated values were arbitrarily set as 100. **(C)** Keratinocytes were transduced with TAT-Rac1^Q61L^ or TAT and lysates immunoprecipitated with anti–β-catenin or anti–E-cadherin antibodies. Alternatively, cells were transfected with pRK5myc-Rac1^Q61L^ (+) or empty vector (−) and immunoprecipitated with anti–E-cadherin antibodies. Precipitated complexes (IP) were probed for the presence of endogenous α-catenin, β-catenin, or E-cadherin. Levels of catenins and cadherin proteins in lysates are shown (input). Statistical significance was analyzed using Student’s *t* test. Scale bar = 20 µm (*n* = 3). *, P < 0.0002.

Upon Rac1 activation, there was no significant difference in the percentage of internalized E-cadherin (vesicular pool) that colocalized with α-catenin, β-catenin, or p120^CTN^, e.g., 70–80% of internalized cadherins (vesicular pool) colocalized with catenins (Fig. 1 C). Similarly, the Pearson coefficient of the cytoplasmic catenins that colocalized with E-cadherin did not differ, albeit it has reduced values at around 0.5 (Fig. 1 D). Reduced Pearson coefficient values may reflect the contribution of the cytosolic pool of catenins associated with distinct partners (i.e., not on vesicles). Unfortunately, the striking Rac1-specific phenotype at junctions (with augmented fluorescence signal in the middle of contacts; Lozano et al., 2008) makes it challenging to compare the relative cadherin-catenin association at junctions with the vesicular pool (Brezovjakova et al., 2019). Similar analyses with E-cadherin immunoprecipitation from transduced (TAT-Rac1^Q61L^) or transfected cells (myc-Rac1^Q61L^) lysates did not show the release of catenins from the complex (Fig. S1 C). Together with previous reports (Akhtar and Hotchin, 2001), our results suggest that activation of Rac1 signaling does not selectively remove catenins from E-cadherin during internalization.

**Figure 2.**
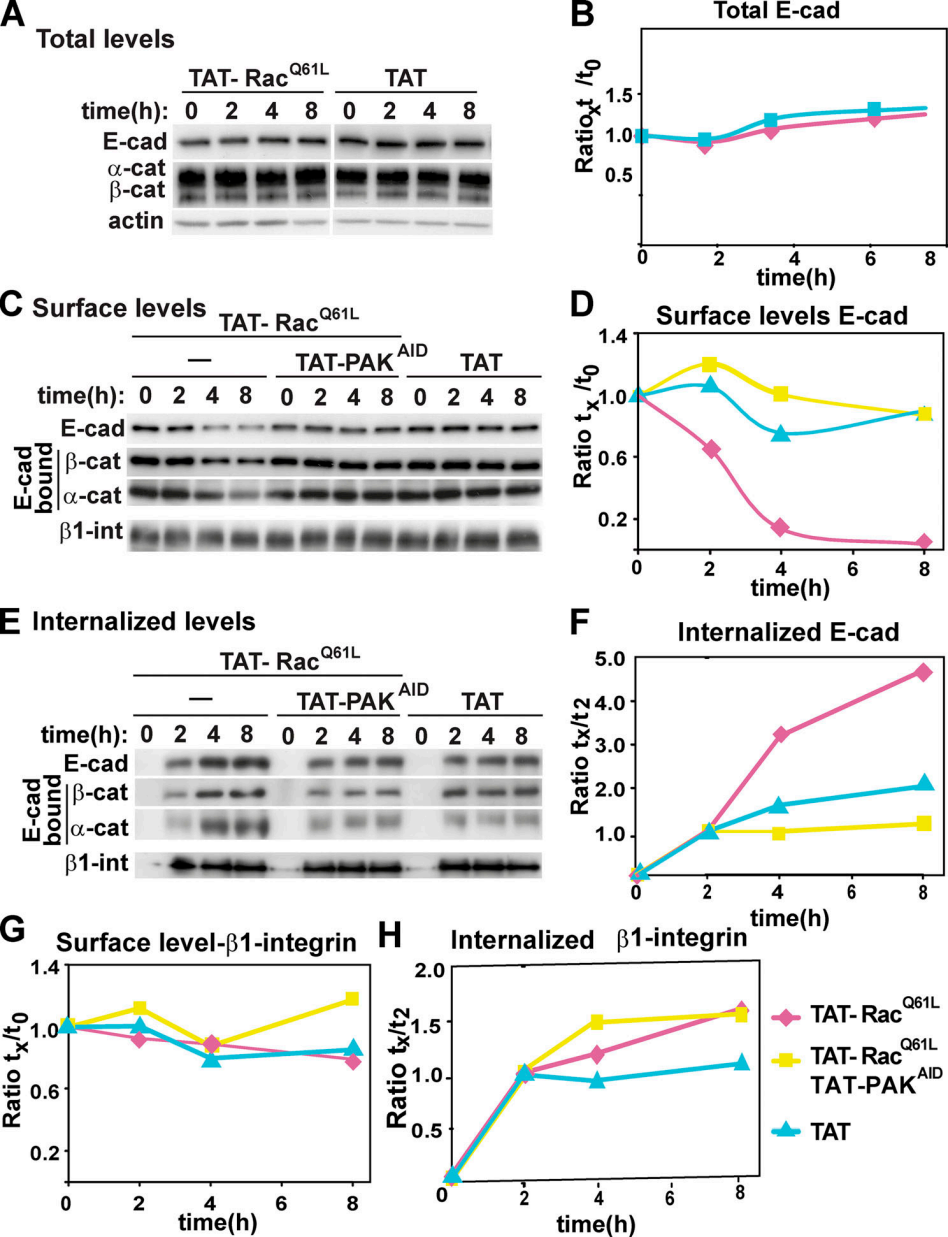
**PAK1 is necessary for internalization of E-cadherin upon Rac^Q61L^ overexpression.** Keratinocytes were treated with cell-permeable TAT or TAT-Rac^Q61L^ in the presence or absence of TAT-PAK^AID^ to inhibit endogenous PAK activation. **(A and B) **Following a time course, cells were surface biotinylated and processed to show total protein levels. **(C–F)** Alternatively, proteins were precipitated with streptavidin to monitor surface levels (C and D) or internalized (E and F) levels of E-cadherin and associated catenins. **(B, D, and F)** Quantification of E-cadherin levels. Representative blots from one independent biological replicate are shown on the left and quantification is shown in graphs on the right (additional replicates are shown in Fig. S2 A). **(G and H)** Quantification of surface (G) and internalized levels (H) of β1-integrin (*n* = 3).

To validate the above results biochemically, cell surface levels and internalized levels were measured following treatment with cell-permeable fusion proteins encoding activated Rac1 (TAT-Rac^Q61L^) and/or the autoinhibitory motif of PAK1 (TAT–autoinhibitory domain of PAK1 [PAK^AID^]; Fig. 2). Controls showed that the TAT-fusion proteins reproduced our results with transfection of active Rac1 and inhibition of PAK1 on junctions (Fig. S1; Lozano et al., 2008). There was no significant degradation of E-cadherin complexes during the timeframe evaluated, as total levels of E-cadherin and catenins were unaltered by adding TAT-Rac1^Q61L^ or control peptide TAT by itself (Fig. 2, A and B). However, in the presence of activated Rac1, E-cadherin surface levels were substantially reduced within 4 h with a corresponding increase in the internalized E-cadherin pool (Fig. 2, C–F). The same profile was observed with α- and β-catenin levels (Fig. 2, C and E), consistent with their co-internalization with E-cadherin shown in immunofluorescence experiments (Fig. 1, B and C). Thus, Rac1 activation does not seem to promote extensive dissociation of catenins before cadherin internalization (Figs. 1 and S1 C).

**Figure S2.**
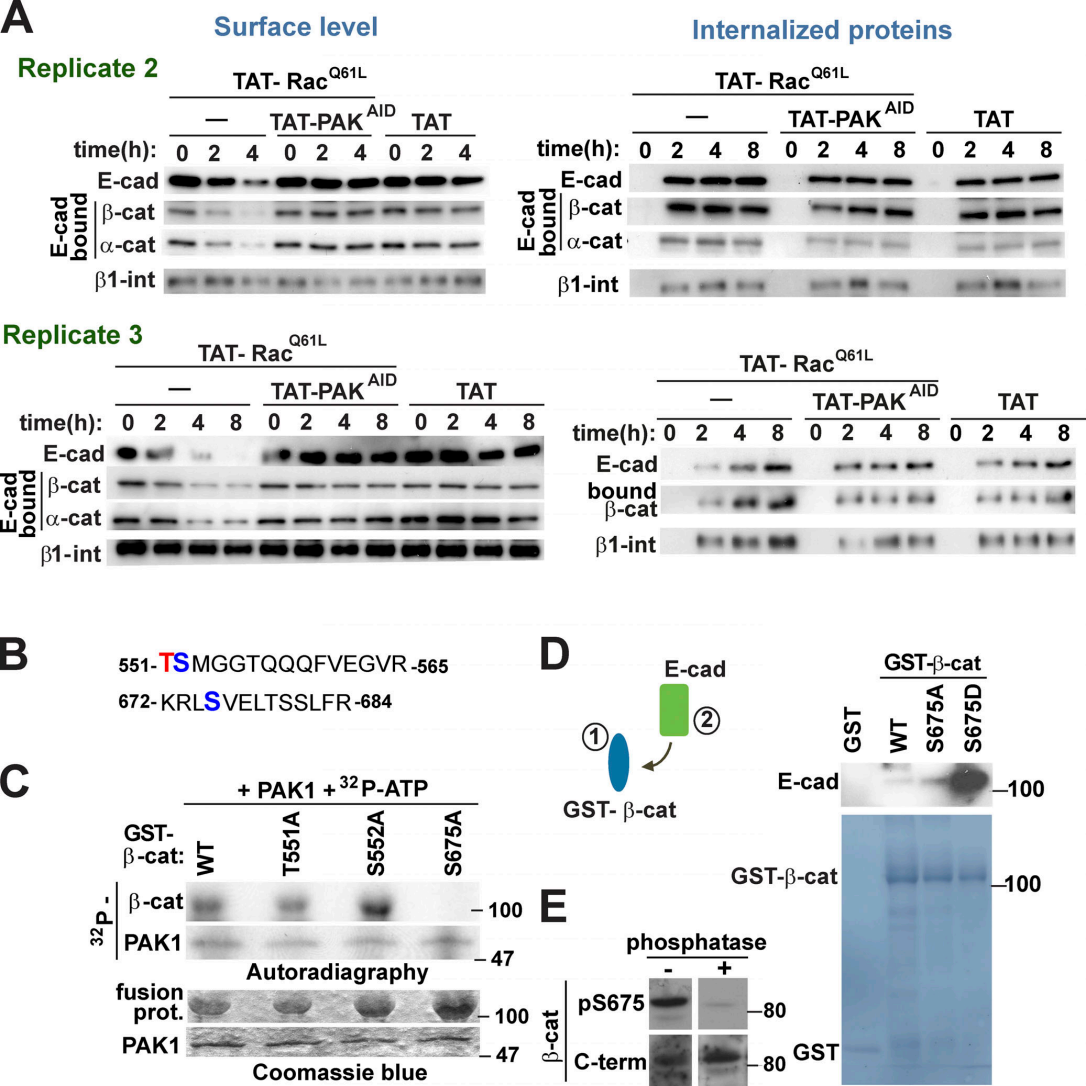
**Internalization assays and PAK1 phosphorylation of β-catenin increases its interaction with cadherin tail.** **(A)** Additional replicates of internalization assays shown in Fig. 2. Keratinocytes were treated with cell-permeable TAT or TAT-Rac^Q61L^ in the presence or absence of TAT-PAK^AID^ to inhibit endogenous PAK activation. Following a time course, cells were surface biotinylated and proteins were precipitated with streptavidin to monitor surface levels (left) or internalized (right) levels of E-cadherin and associated catenins. As a control, surface and internalized β1-integrin levels were measured. **(B)** Peptides identified by mass spectrometry as potential serine phosphorylation sites for PAK1 on β-catenin following in vitro phosphorylation assays. Putative threonine (T) is highlighted in red font and serines (S) are shown in blue font. **(C)** Identification of PAK1 phosphorylation site. In vitro PAK phosphorylation assay using GST–β-catenin WT and alanine mutants (T551A, S552A, or S675A). **(D)** Association of E-cadherin cytoplasmic tail with GST-tagged β-catenin WT and mutants (nonphosphorylatable β-cat^S675A^ or phosphomimetic β-cat^S675D^) using pull-down assays. **(E)** Specificity of antibody against phosphorylated β-catenin (pS675). Keratinocyte lysates were incubated in the presence or absence of calf intestinal phosphatase, and Western blots were probed with anti–β-catenin antibodies raised against the phosphorylated form (pS675) or the C-terminal region (C-term; *n* = 3).

When endogenous PAK1 activation by Rac1 was inhibited (TAT-Rac^Q61L^ + TAT-PAK^AID^), a decrease in surface levels of cadherin complexes (Fig. 2 C) and co-internalization of E-cadherin and catenins was prevented (Fig. 2, E and F; see also Fig. S2 A). These effects were specific for E-cadherin, as surface and internalized levels of β1 integrins were not perturbed to similar extent as E-cadherin (Fig. 2, G and H). We concluded that, upon constitutive activation of Rac1 and PAK1, E-cadherin complexes are internalized, but not degraded.

### PAK1 phosphorylates cadherin tail and β-catenin to strengthen their interaction

**Figure 3.**
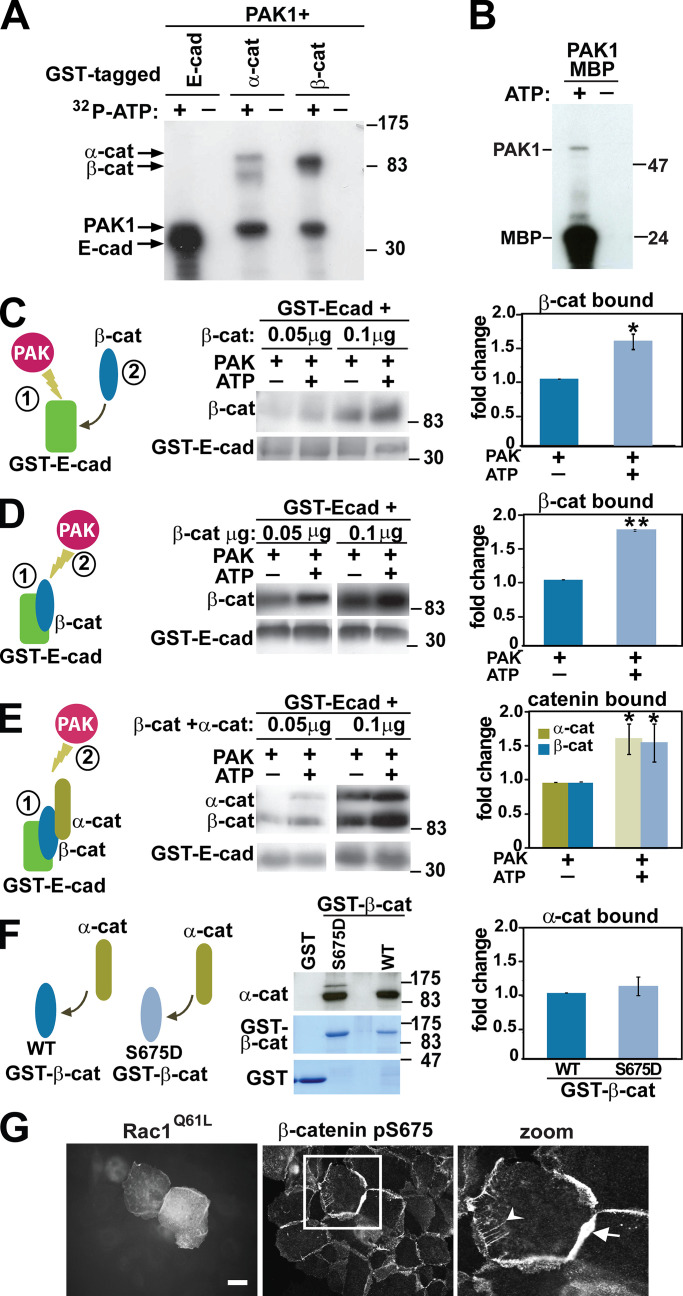
**PAK1 phosphorylation strengthens the interaction between E-cadherin and catenins.** **(A and B)** In vitro kinase assay using different GST-tagged proteins as substrates and purified PAK1 kinase domain in the presence or absence of ^32^P-ATP. Images show radioactively phosphorylated proteins. PAK1 autophosphorylation (A and B) and MBP (B) are used as internal controls. **(C–F)** In vitro reconstitution of cadherin complex with or without PAK1 phosphorylation. Diagrams on the left represent proteins used and numbers show the order of phosphorylation and binding. Blots show the amount of cleaved catenins that interact with GST–E-cadherin cytoplasmic tail (C–E) or GST–β-catenin (F) under each condition. Graphs on the right show quantification of the protein interaction, and values are expressed relative to controls (nonphosphorylated proteins). **(G)** Rac^Q61L^-transfected keratinocytes were stained with β-catenin pS675 antibody. Inset is a zoom of cell highlighted by the white square. Arrow shows enlargement of junctional staining, and arrowhead points to labeled intracellular tubular structures. Scale bar = 10 µm or 4 µm (zoom; *n* = 3). Samples were analyzed with *t* test, and error bars represent SD. *, P < 0.05; **, P = 0.0002.

We initially addressed the potential destabilization of cadherin complexes by phosphorylation. In in vitro kinase assays, purified PAK1 kinase phosphorylated E-cadherin tail and β-catenin fusion proteins and the positive control maltose-binding protein (MBP; Fig. 3, A and B). A weaker phosphorylation of α-catenin was also observed but not investigated further here. It is feasible that the identified phosphorylation of cadherin tail or β-catenin by PAK1 may contribute to the release of cadherin complexes from junctions.

In vitro reconstitution assays were set up to evaluate the modulation of cadherin-catenin interaction by PAK1 phosphorylation. Stronger interaction of β-catenin with phosphorylated GST–E-cadherin tail was observed (Fig. 3 C). In addition, phosphorylation of preassembled E-cadherin complexes by PAK1 also enhanced the presence of catenins in the precipitated samples (Fig. 3, D and E). Contrary to expectations, PAK1 phosphorylation of E-cadherin tail does not reduce β-catenin association. Instead, phosphorylation promotes a more stable complex in vitro (Fig. 3, C and D). Consistent with our findings, no changes in endogenous cadherin complex stoichiometry were observed by various precipitation approaches (Fig. S1 C), despite strong disruption of cadherin-mediated adhesion.

Putative PAK1 phosphorylation sites on β-catenin were predicted by mass spectrometry at Thr551, Ser552, or Ser675 (Fig. S2 B), in line with previous studies (Rennefahrt et al., 2007; Taurin et al., 2006). The mutant S675A was unable to be phosphorylated by PAK1 in vitro, suggesting that β-catenin is phosphorylated at a single site by PAK1 (Fig. S2 C) and confirming previous findings (Zhu et al., 2012). PKA, PAK1, and PAK4 phosphorylate β-catenin at Ser675, promoting stabilization and increased transcription of β-catenin responsive genes in cell lines (Hino et al., 2005; Li et al., 2012; Selamat et al., 2015; Zhu et al., 2012). However, we were unable to demonstrate higher transcription levels of β-catenin responsive genes in normal keratinocytes (data not shown).

The impact of β-catenin Ser675 phosphorylation on cadherin adhesion function or stability has not been determined. In vitro complex reconstitution showed that phosphomimetic β-catenin (S675D) interacted with recombinant E-cadherin tail more efficiently than WT or nonphosphorylatable mutant β-catenin (S675A; Fig. S2 D). However, a phophomimetic β-catenin mutant did not show stronger binding for α-catenin (Fig. 3 F). These results indicate that PAK1 phosphorylation of β-catenin enhances the affinity for cadherin tail, but not α-catenin.

Using an antibody against phosphorylated β-catenin at Ser675, a pool of endogenous phosphorylated β-catenin was detected at junctions during homeostasis and at stable cell-cell contacts (Fig. 3 G). In Rac1-expressing cells, phosphorylated β-catenin was also present at disrupted junctions and in intracellular tubules and vesicles (Fig. 3 G, zoom). Taken together, these data strongly indicate that E-cadherin is in complex with catenins inside cells and that PAK1 phosphorylation unexpectedly enhances the association between cadherin and β-catenin.

### Following Rac1 activation, E-cadherin is internalized via micropinocytosis

**Figure 4.**
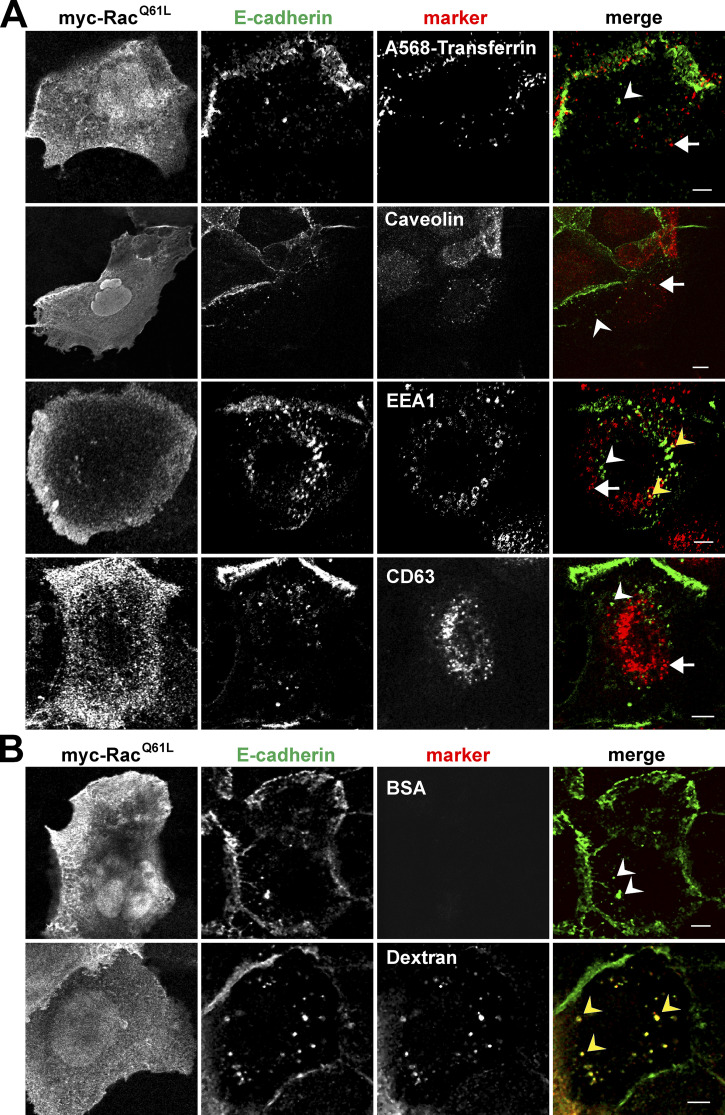
**Rac1 activation promotes E-cadherin internalization via fluid uptake.** **(A)** Keratinocytes expressing activated Rac1 (Rac1^Q61L^) were fixed and stained with antibodies against the tag, E-cadherin, and various intracellular markers to visualize caveolae (caveolin), early endosomes (EEA1), or late endosomes/lysosomes (CD63). **(A and B)** Alternatively, keratinocytes expressing activated Rac1 were incubated with Alexa Fluor 568-Transferrin (A; A568-transferrin) to detect clathrin-dependent internalized vesicles, Texas Red-BSA to label macropinocytosis, or Texas Red-Dextran to show micropinocytosis (B) for 30 min. After incubation, cells were stained and imaged using confocal microscopy. Merged files are shown in the last column. White arrows show intracellular marker staining, white arrowheads show cadherin vesicular staining, and yellow arrowheads show colocalization of E-cadherin with intracellular marker. Images are representative of three independent experiments. Scale bars = 2 µm.

The unexpected finding that E-cadherin is internalized by Rac1 activation without significant dissociation of catenins in primary keratinocytes (Figs. 1, 2, and 3) is consistent with data from tumor cell lines that the disruption of cell–cell adhesion may not use the classical internalization routes (Akhtar and Hotchin, 2001). To identify the E-cadherin vesicular pool in the cytoplasm, we costained samples expressing Rac1 with E-cadherin antibodies and a panel of intracellular markers (Fig. 4). In spite of considerable perturbation of junctions, there was no substantial overlap between E-cadherin–containing vesicles and markers of clathrin- or caveolin-dependent internalization (transferrin or caveolin, respectively), early endosomes, or late endosome/lysosome compartment (CD63; Fig. 4 A). By feeding cells with fluorescently labeled BSA, we could also exclude macropinocytosis, a typical membrane turnover process induced by Rac1 and PAK1 (Fig. 4 B; Dharmawardhane et al., 2000). In contrast, treating keratinocytes with a smaller fluorescent compound (dextran; 10 kD), substantial colocalization with E-cadherin on small vesicles was observed (Fig. 4 B). The micropinosome compartment and its intracellular trafficking routes are poorly defined. Nevertheless, our data indicate that, rather than macropinocytosis as shown in keratinocyte tumor cell lines (Akhtar et al., 2000), Rac1 activation promotes E-cadherin internalization via fluid uptake that is likely to be micropinocytosis.

### PAK1 phosphorylates and modulates RabGDIβ function

Our results suggest that the junction defects caused by active Rac1 in normal keratinocytes cannot be explained by a PAK1-driven, looser association between cadherin and catenins or promotion of β-catenin nuclear function (data not shown). We reasoned that Rac1 signaling could modulate E-cadherin complex internalization by targeting the endocytic machinery. In mammalian cells, in addition to macropinocytosis (Dharmawardhane et al., 2000), PAK1 participation in intracellular trafficking has been reported with internalization (Karjalainen et al., 2008) and glucose uptake (Tunduguru et al., 2017); however, its endocytic roles are generally thought to occur via PAK1-dependent cytoskeletal reorganization rather than a direct modulation of the trafficking machinery.

PAK1 may dissociate cell-cell junctions by regulating E-cadherin intracellular transport rather than complex stability. We speculated that PAK1 may phosphorylate RabGDI, the regulator of Rab small GTPase retrieval and delivery to different intracellular compartments (Shinde and Maddika, 2018). The RabGDI counterpart, RhoGDI, is a key regulator of Rho GTPases. PAK1 phosphorylates RhoGDI at Ser101 and Ser174, which promotes its dissociation from Rac1, but not RhoA (Ard et al., 2012; DerMardirossian et al., 2004). Similar phosphorylation of RabGDI could modulate their affinity for different Rabs and thus target specific trafficking pathways.

**Figure 5.**
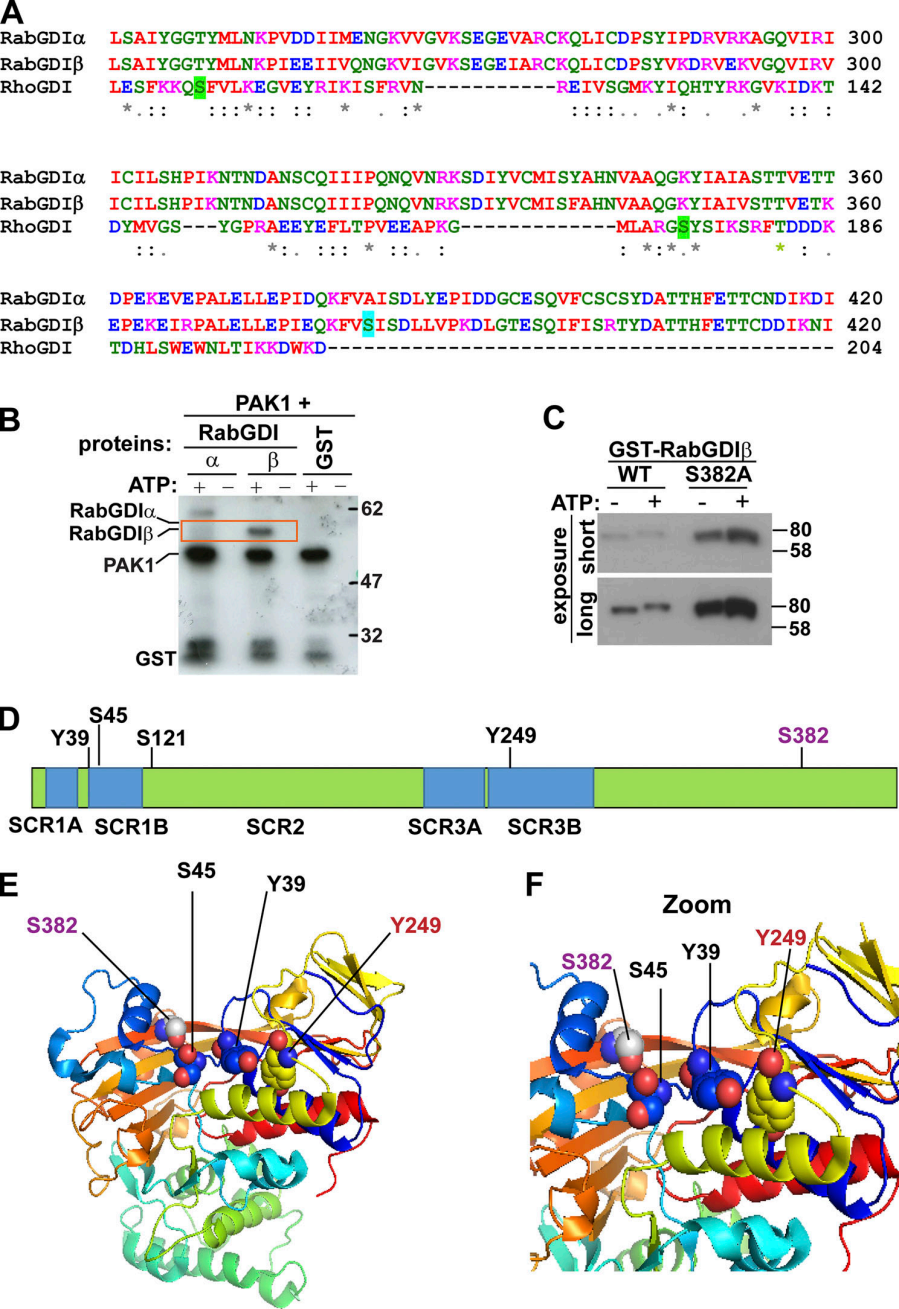
**PAK1 phosphorylates RabGDIβ.** **(A)** Sequence alignment of RabGDIα, RabGDIβ, and RhoGDI. PAK1 phosphorylation sites on RhoGDI are highlighted in green. Based on PAK1 phosphorylation motifs of known substrates (Fig. S3), putative phosphorylated site by PAK1 on RabGDIβ is highlighted (light blue). **(B)** In vitro kinase assay using purified proteins incubated with PAK1 kinase with and without radioactive ATP: RabGDIα, RabGDIβ, and MBP (positive control) or GST (negative control). PAK1 autophosphorylation is shown. **(C)** RabGDIβ WT or nonphosphorylatable mutant (S382A) were phosphorylated in vitro as described in B and separated using a Phostag gel to show mobility retardation of phosphorylated proteins. **(D)** Schematic diagram of RabGDIα/β domain structure showing shared conserved domains (sequence conserved region [SCR]; Schalk et al., 1996) and phosphorylated amino acids on RabGDIα or RabGDIβ known to modulate Rab binding. **(E and F)** Crystal structure of RabGDI-α (Protein Data Bank accession no. 1GND) mapping the different phosphorylation sites. Phosphorylated residue identified in RabGDIβ is shown in purple font (S382). **(F)** Zoom of the Rab binding platform on RabGDIα shows the proximity of residues identified in this work and in the literature.

RhoGDI was aligned with RabGDI sequences to determine whether the PAK1-phosphorylated amino acids are conserved in RabGDI (Fig. 5 A, highlighted in green). The main RhoGDI phosphorylated site by PAK1—Ser174—was replaced by a lysine (position 357) in RabGDIα (brain specific) and RabGDIβ (ubiquitously expressed). RhoGDI Ser101 corresponded to Thr248 found in both RabGDIα and RabGDIβ and could be a site for PAK1 phosphorylation. However, in vitro kinase assay with purified proteins showed that PAK1 phosphorylated RabGDIβ, but not RabGDIα (Fig. 5 B). Such exclusive phosphorylation of RabGDIβ (Fig. 5 B) suggests that it is unlikely that T248 is the PAK1 site phosphorylated on RabGDIβ and that alternative sites must exist.

**Figure S3.**
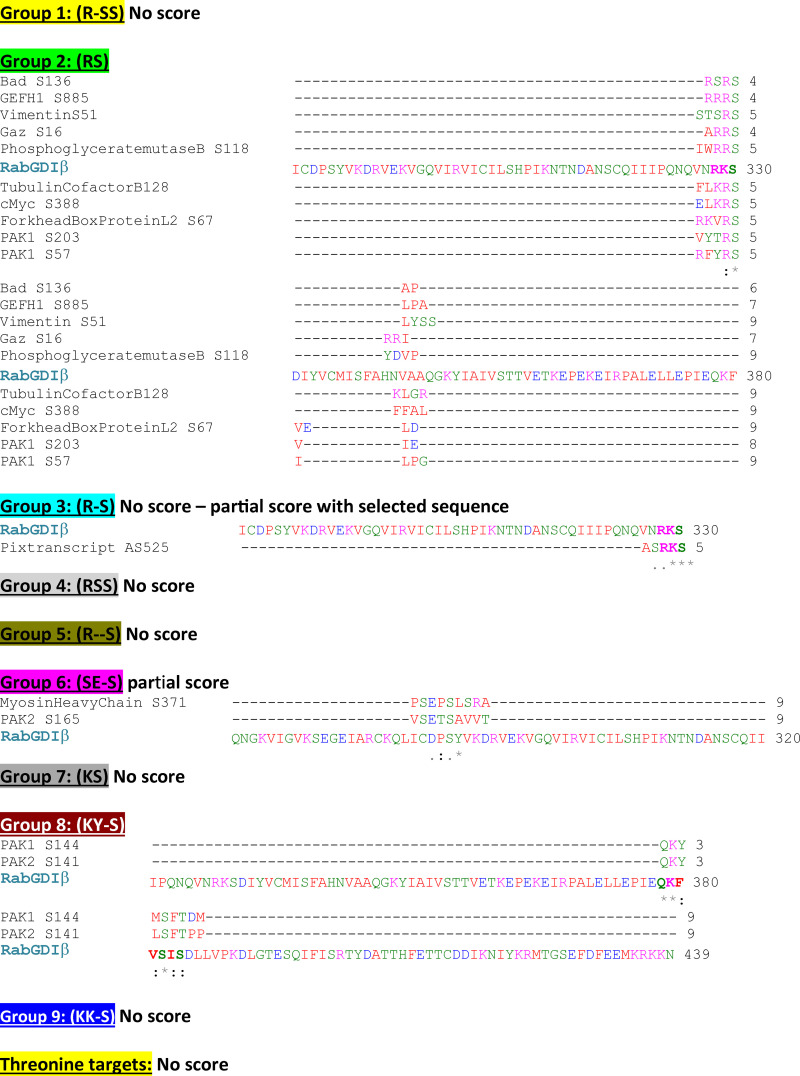
**Search for putative phosphorylation sites in RabGDIβ.** PAK1 is a promiscuous kinase that phosphorylates a variety of cellular substrates. As a PAK1 phosphorylation motif has not been defined on RabGDI proteins, known PAK1 substrates were grouped according to the phospho-site (nine separate groups). Substrate names are shown on the left of each row. RabGDIβ sequence was blasted to identify potential homologies. Two putative phosphorylation sites on RabGDIβ were identified at positions S285 and S330 compared with Group 2 (RS) and Group 3 (R-S) substrates. Another potential site was identified at position 382 compared with Group 8 (KY-S). Serine 382 is not found in RabGDIα and thus it is likely the RabGDIβ amino acid phosphorylated by PAK1. No other putative phosphorylation sites were identified compared with other groups.

**Figure S4.**
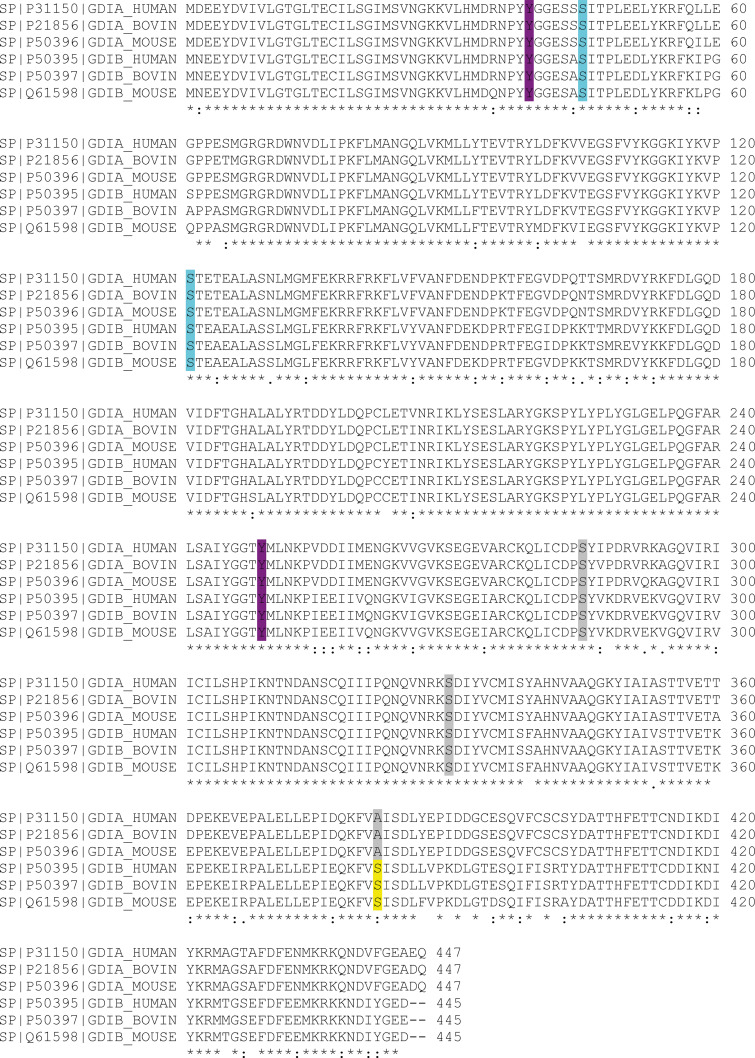
**Alignment of RabGDIα and RabGDIβ from human, bovine, and mouse species.** Alignment of full-length amino acid sequences of RabGDIα (GDIA) and RabGDIβ (GDIB) was done using UniProt Align. Sequences from different species are shown with the access number on the left of each row. Conservation of putative phosphorylation sites across different genes is highlighted as follows. Highlighted amino acid residues: Tyrosine (magenta), serine (cyan), or predicted phosphorylation sites by alignment with PAK substrate motifs (gray). Serine 382 is found exclusively in RabGDIβ. See text for more details.

To provide insights into additional residues that PAK1 could phosphorylate on RabGDIβ, we aligned RabGDIβ sequence with known PAK1 substrates. PAK1 substrates were grouped according to their consensus phosphorylated motifs and aligned with RabGDIβ (Fig. S3). From this analysis, putative PAK1 phosphorylation sites could be Ser285, Ser330, and Ser382 (Fig. S3). These amino acids are conserved among different species (Fig. S4, highlighted in gray). Two of the predicted RabGDIβ phospho-sites by PAK1 (Ser285 and Ser330) are also conserved in RabGDIα and were thus excluded from our consideration.

The predicted phospho-site (Ser382) is found only in RabGDIβ and thus the likely site to be phosphorylated by PAK1 (Fig. S4, highlighted in yellow; Fig. 5 A, highlighted in cyan). Following in vitro kinase assays using PAK1 kinase, WT RabGDIβ, or a mutant unable to be phosphorylated (S382A; Fig. 5 C) were run on Phostag gels, which is a qualitative assessment using retardation of phosphorylated proteins as a readout. A mobility shift was observed with WT RabGDIβ, consistent with addition of a negative charge by phosphorylation. Mutation to alanine at residue 382 (S382A) abolished the shift, suggesting that this is the site where PAK1 phosphorylated RabGDIβ (Fig. 5 C).

Serine 382 localizes at the C terminus of RabGDIβ, outside the RabGDI sequence conserved regions (Fig. 5 D; Luan et al., 2000; Schalk et al., 1996; Wu et al., 1998). This residue is also away from known amino acids that modulate Rab binding on RabGDIβ (i.e., Tyr39 and Tyr249; Shisheva et al., 1999) or RabGDIα (Ser121 and Ser45; Cavalli et al., 2001). Yet, in the 3D structure, all of these residues are in close proximity within the predicted Rab binding platform (Fig. 5, E and F; Luan et al., 2000). These analyses indicate that PAK1 phosphorylation at Ser382 may regulate the interaction between RabGDIβ and Rabs.

**Figure S5.**
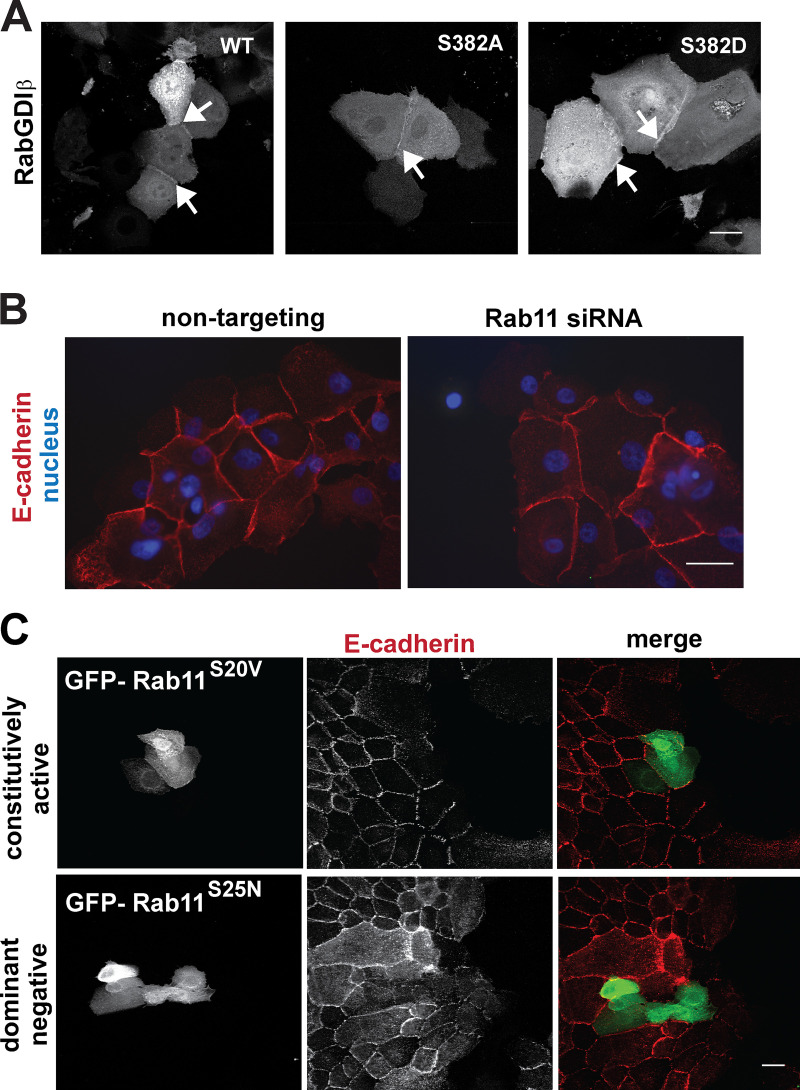
**Localization of RabGDIβ or Rab11 mutants at junctions does not impair cell–cell contacts.** **(A)** Keratinocytes were transfected EGFP-RabGDIβ WT or mutants nonphosphorylatable (S382A) or phosphomimetic (S382D). After overnight incubation, cells were fixed and confocal images collected. Arrows point to RabGDIβ localization at junctions. **(B)** Keratinocytes were transfected with Rab11 siRNA or nontargeting control oligos, fixed, and stained for E-cadherin and nuclei. **(C)** Keratinocytes were microinjected with constitutively active Rab11 (S20V) or dominant-negative Rab11 (S25N). Cells were fixed, stained for E-cadherin, and imaged in a confocal microscope to detect E-cadherin and the GFP tag. Scale bar = 20 µm (A and B) or 40 µm (C).

**Figure 6.**
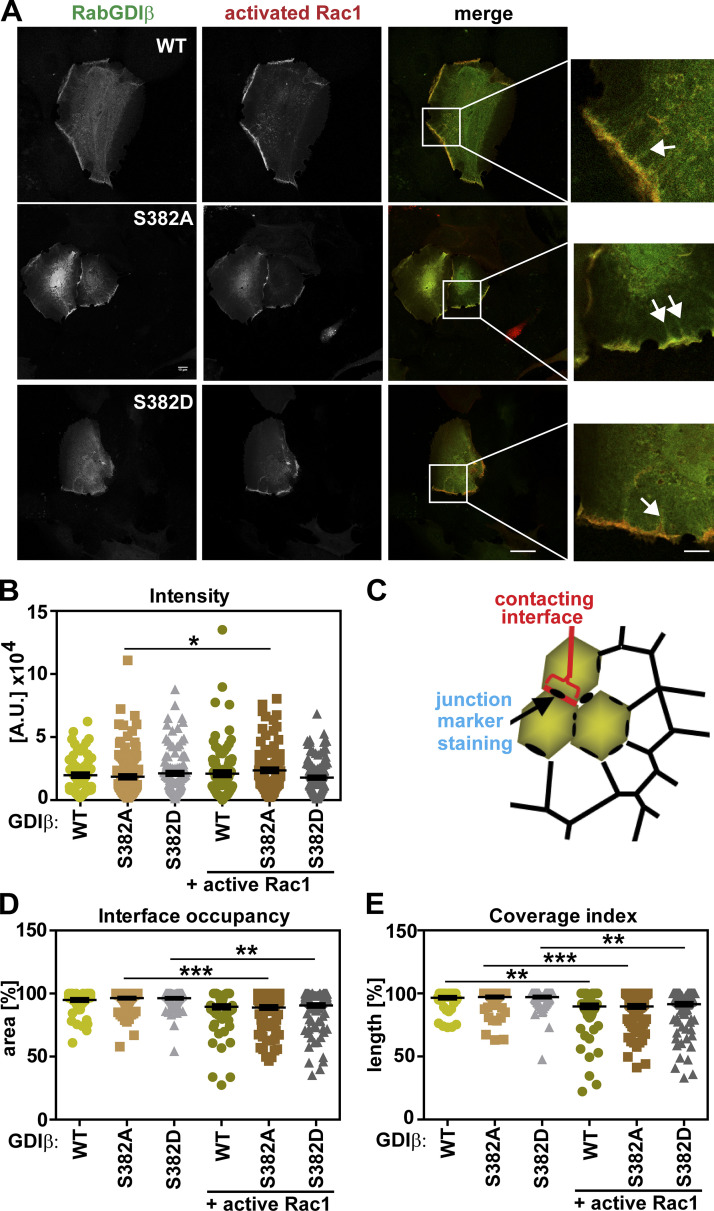
**RabGDIβ localizes at cell–cell contacts and cytoplasm of keratinocytes.** **(A)** pEGFP-RabGDIβ WT and mutants nonphosphorylatable (S382A) or phosphomimetic (S382D) were expressed in keratinocytes in the presence of activated Rac1 (mRFP-Rac^Q61L^; see also Fig. S5 A). Cells were fixed and representative confocal images are shown. **(B)** Staining levels of RabGDIβ at junctions between coexpressing cells were quantified by measuring the total intensity of exogenous proteins at cell–cell contacts. **(C)** Diagram showing the quantified junctional areas: Contacting membrane between neighboring cells and the staining fragments of the junction marker. **(D and E)** Graphs show the area of the contacting interface that contains RabGDIβ pixels (D; interface occupancy) and the length of RabGDIβ fluorescence that covers the contacting interface length (E; coverage index). Scale bar = 20 µm or 7 µm (zoom). Arrows point to tubular structures at cell–cell contacts where Rac1 and RabGDIβ colocalize. Statistical analyses performed with Kruskal-Wallis with Dunn's multiple comparison test; error bars represent SEM. *, P < 0.05; **, P < 0.01; ***, P < 0.001.

The localization of RabGDI in cells is unknown. To investigate whether PAK1 phosphorylation alters RabGDIβ localization, pEGFP-RabGDIβ WT and mutants (S382A or S382D) were expressed in keratinocytes by themselves and showed cytoplasmic distribution and an unexpected junctional localization (Fig. S5 A, arrows). In addition to the predicted cytoplasmic localization, in the presence of activated Rac1 (pRFP-Rac1^Q61L^; Fig. 6 A), all RabGDIβ constructs colocalized with Rac1 at junctions and at tubular structures originating from cell–cell contacts (Fig. 6 A, merge zoom, arrows). When the intensity at junctions was quantified, there were no differences in RabGDIβ levels at steady state (Fig. 6 B). Following Rac1 activation, a small but significant increase in nonphosphorylatable RabGDIβ (S382A) intensity at cell–cell contacts was observed (Fig. 6 B). We next evaluated the potential influence of Rac1 on the localization of pEGFP-RabGDIβ fluorescence at the contacting interface between neighboring cells (Fig. 6, C–E). Rac1 activation reduced the number of pixels of both RabGDIβ mutants at the contacting interface area (Fig. 6 D), while there was no significant change in the levels of WT RabGDIβ. When coverage index was considered (i.e., RabGDIβ pixel length that covered the contacting interface length), there was a consistent reduction of all constructs at junctions (Fig. 6 E). We concluded that RabGDIβ phosphorylation does not alter its junctional localization at steady state or in response to Rac1 activation (Fig. 6, B and D).

### Rac1 signaling activates Rab11

**Figure 7.**
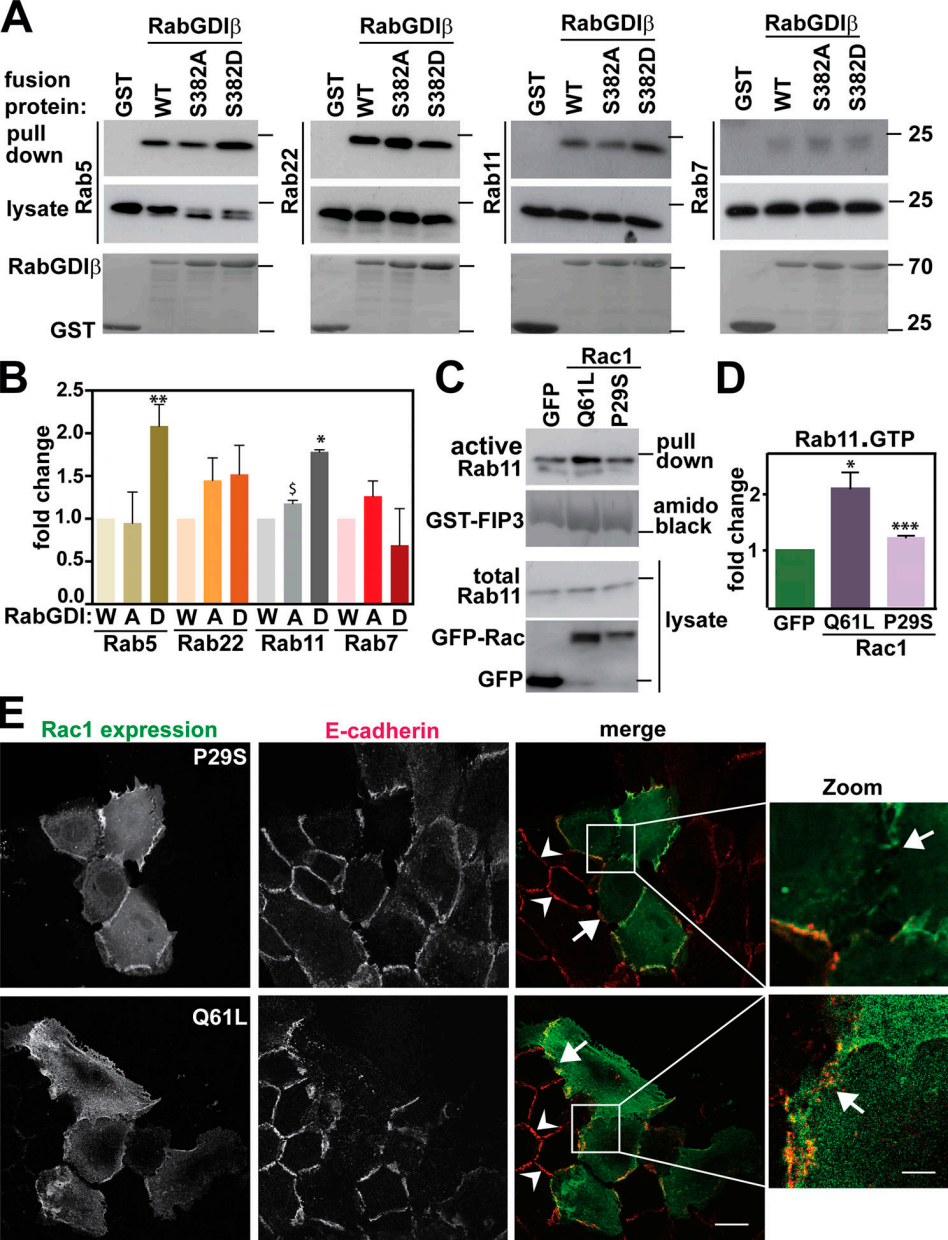
**Phosphomimetic RabGDIβ differentially interacts with Rab5 and Rab11.** **(A)** GST-RabGDIβ WT, nonphosphorylatable (S382A), or phosphomimetic (S382D) mutant was incubated with keratinocyte lysates and coprecipitated Rabs identified with specific antibodies denoted on the left of each panel. Lysate samples show total levels of Rabs. GST was used as control and fusion proteins are shown in bottom panels. **(B)** Quantification of RabGDIβ-associated Rabs as shown in A. The amount of different endogenous Rabs coprecipitated with GST-RabGDIβ (pull-down) was quantified and normalized to the total amount of Rab (lysates). Values were expressed as fold change from each Rab association with WT RabGDIβ. **(C)** Levels of endogenous active Rab11 (Rab11·GTP, pull-down) were detected from lysates of cells transfected with GFP-Rac^Q61L^ or GFP-Rac^P29S^ using GST-FIP3 (amido black). Levels of Rab11 were detected with anti-Rab11 antibody, and levels of GFP-Rac1 or GFP in lysates were shown with anti-GFP antibody. Molecular weight marker (25 kD) is shown on the right of blots. **(D)** Quantification of Rab11-FIP3 pull-down. Intensity of Rab11 bands associated with GST-FIP3 (active pool) was quantified and expressed as percentage of the levels of endogenous Rab11 found in lysates (total Rab11). Basal levels of active Rab11 (GFP-expressing cells) were used as a control and values arbitrarily set at 1. **(E)** Keratinocytes were transfected with pEGFP-Rac^P29S^ (top) or pEGFP-Rac^Q61L^ (bottom) and stained for E-cadherin. Statistical analyses were performed using Student’s *t* test. Arrowheads show intact junctions and arrows point to disrupted junctions. Scale bar = 20 µm or 7 µm (zoom). Representative images are shown in A, C, and E. Graphs show mean values and SD (*n* = 3). *, P < 0.02; ^$^, P = 0.043; **, P = 0.05; ***, P = 0.006.

The functional significance of RabGDIβ phosphorylation was tested on its ability to interact with different Rabs (Fig. 7). GST or different GST-RabGDIβ fusion proteins were incubated with keratinocyte lysates at steady state and precipitated Rabs detected by Western blots (Fig. 7 A). Phosphomimetic RabGDI mutant (S382D) showed a significant increase in interaction with endogenous Rab5 and Rab11 compared with WT or nonphosphorylated forms (Fig. 7 B). No differences were detected in the association with Rab7 or Rab22, indicating that distinct Rabs can be discriminated by phosphomimetic RabGDIβ. Thus, the differential binding of RabGDIβ to Rabs may suggest that the retrieval of selected Rabs from membranes is modified by phosphorylation at Ser382.

We focused on the potential role of Rab11 in the disassembly of junctions caused by Rac1. PAK1 was not able to phosphorylate Rab11 in vitro (data not shown), but it is feasible that Rac1 signaling could modulate Rab11 activity. For these experiments, we tested Rac1 mutants containing the constitutively active mutation Q61L or the tumor-derived, fast-cycling mutation P29S, highly prevalent in melanomas (Porter et al., 2016). Expression of these mutants is predicted to mimic WT Rac1 gene amplification or mRNA overexpression in tumors (Porter et al., 2016).

Using GST-FIP3 pull-down assays (Franco et al., 2014), Rab11 was activated following expression of either Rac1 mutant (Fig. 7, C and D). Furthermore, the oncogenic Rac1^P29S^ was also able to disrupt cell-cell contacts, although less efficiently than with Rac^Q61L^ (Fig. 7 E). We concluded that, rather than inactivating Rab11, Rac1 mutants promote Rab11 activation. We surmise that it is likely that Rab11-dependent transport participates in cadherin adhesion disruption.

**Figure 8.**
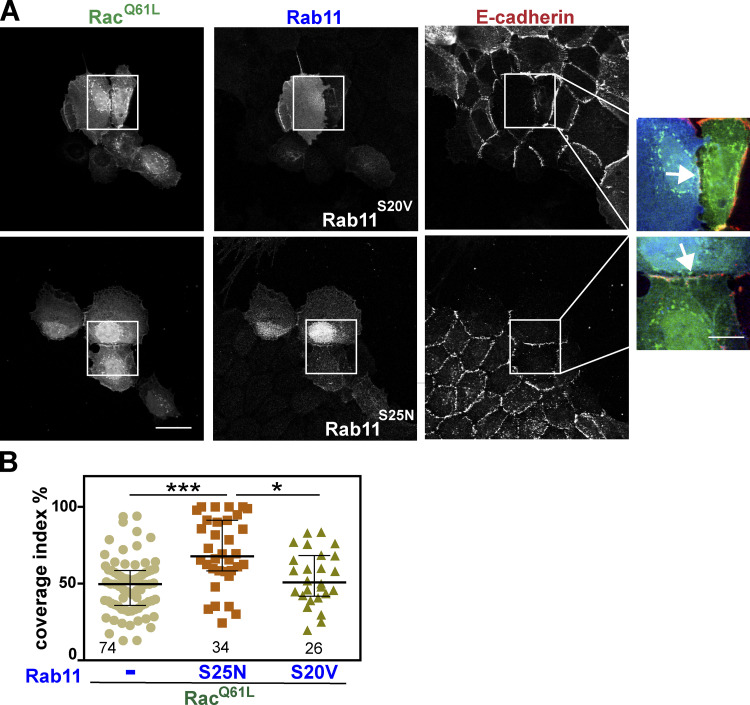
**Rab11 activation is necessary for the disruptive effect of activated Rac1 on junctions.** **(A)** Keratinocytes were microinjected with activated Rac1 (Rac1^Q61L^) and constitutively active (Rab11^S20V^) or dominant-negative Rab11 (Rab11^S25N^) to prevent activation of endogenous Rab11. Following incubation for 6 h, cells were fixed and stained for E-cadherin. Zoom shows merged images amplified from white box regions. Scale bar = 20 µm or 7 µm (zoom). Arrows point to junctions perturbed by expression of Rac1^Q61L^. **(B)** Quantification of Rac1 defects on junctions using the parameter coverage index. Numbers below each sample means the number of junctions analyzed. Statistics were performed using Kruskal-Wallis with Dunn's multiple comparison test; error bars represent SEM (*n* = 3, Rab11^S25N^; or *n* = 2, Rab11^S20V^). *, P < 0.05; ***, P < 0.001.

**Figure 9.**
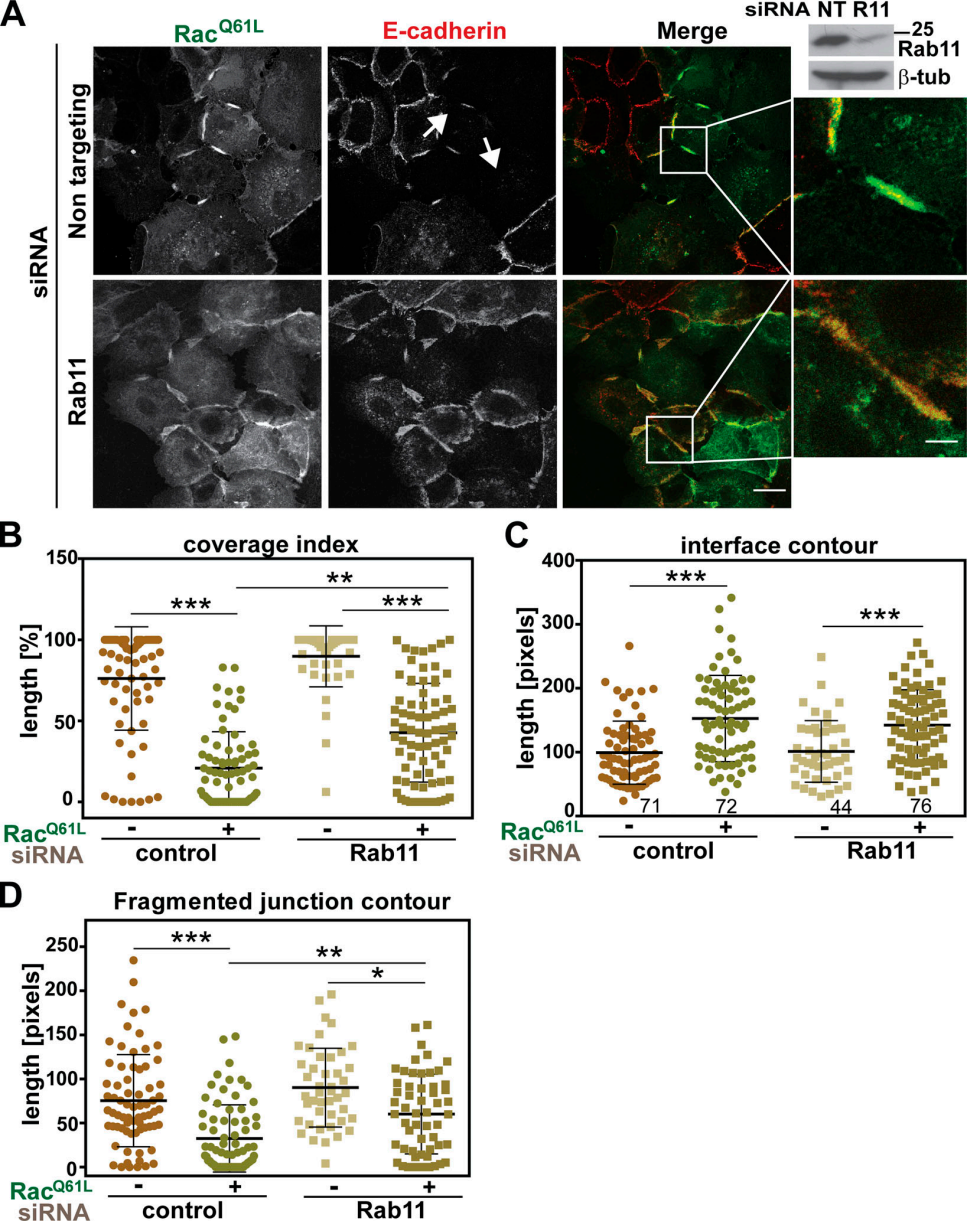
**Depletion of Rab11 partially rescues activated Rac1-dependent junction disruption.** **(A)** Keratinocytes were treated with Rab11 siRNA or control oligos (nontargeting [NT]) and transfected with activated Rac1 (Rac1^Q61L^). Representative Western blot confirms reduction of Rab11 levels for each experiment; β-tubulin is used a loading control. Zoom shows images amplified from white box regions. Arrows point to junctions perturbed by expression of Rac1^Q61L^. **(B–D)** Quantification of the defects caused by Rac1 activation on junctions: Coverage index (B), the length of contacting membranes between neighboring cells (C; interface contour), and the length of fragments of E-cadherin staining along junctions (D; fragmented junction contour). Scale bar = 20 µm or 7 µm (zoom). Statistics were performed using Kruskal-Wallis with Dunn's multiple comparison test; error bars represent SEM (*n* = 2). Number of junctions analyzed for each sample for all quantifications (B–D) is written inside the graph in C. *, P < 0.05; **, P < 0.01; ***, P < 0.001.

To test whether Rab11 function is necessary downstream of Rac1 activation, we used two approaches: Expression of Rab11 mutants (Fig. 8) or depletion of endogenous Rab11 (Fig. 9). Following expression of constitutively active Rab11 (S20V) or dominant-negative Rab11 (S25N), samples were stained with anti–E-cadherin antibodies (Fig. 8 A) and the percentage of contacting interface length covered by cadherin staining was quantified (Fig. 8 B). In the presence of activated Rac1, preventing endogenous Rab11 activation with the dominant-negative Rab11^S25N^ partially rescued the perturbation of cell–cell contacts (Fig. 8 B). In contrast, coexpression with GTP-locked Rab11 (Rab11^S20V^) had no effect. These results are consistent with the interpretation that activation of endogenous Rab11 by Rac1 (Fig. 7, C and D) is required for junction disruption.

We confirmed the above data by depletion of endogenous Rab11 in the presence of Rac1 activation (Fig. 9). Rab11 depletion was confirmed for each experiment (Fig. 9 A) and collected images were quantified. There was no significant difference in the effect of Rab11 siRNA on the coverage of E-cadherin staining at steady state without Rac1 expression (Fig. 9 B). When Rac1 was activated, endogenous Rab11 depletion partially rescued Rac1-dependent junction disruption compared with samples treated with control siRNA oligos (Fig. 9 B). The cellular effect of Rac1 activation was twofold: An increase in the length of contacting interface between cells as cells flatten out (Fig. 9 C), with a concomitant reduction in the length of E-cadherin staining (Fig. 9 D). Rab11 depletion partially rescued the reduction of E-cadherin length at contact sites caused by Rac1 (Fig. 9 D) but had no effect of the length of contacting interface (Fig. 9 C).

The above experiments suggest the involvement of Rab11 downstream of active Rac1 in junction stability; however, modulation of Rab11 function, per se, did not mimic the disruption phenotype by Rac1 activation (Fig. S5 B). Expression of constitutively active (Rab11^S20V^) or dominant-negative (Rab11^S25N^) Rab11 did not significantly alter cell shape, junction morphology, and E-cadherin staining levels (Fig. S5 C). Similarly, Rab11 depletion, per se, did not interfere with the increase in the interface contour of E-cadherin staining in the absence of Rac1 expression (Fig. 9 C). Taken together, our data indicate that Rab11 is necessary, but not essential, for E-cadherin stability at steady state, consistent with a slow turnover and high stability of mature E-cadherin complexes at junctions. In the context of a disruption stimulus, such as Rac1 activation, we surmise that cooperation of distinct pathways promotes the characteristic phenotype of junction disruption (Braga et al., 2000), including activation of PAK1 (Lozano et al., 2008), and changes in RabGDIβ interaction profile and its integration with Rab11 activation (this work).

## Discussion

This work contributes to our conceptual understanding of the crosstalk between Rac1 and intracellular trafficking in ways previously unappreciated. We identify a core process by which PAK1 directly controls Rab function, which underpins broader mechanisms during membrane remodeling in adhesion, pinocytosis, lamellae protrusion, and motility. Uncontrolled Rac1 activation perturbs cell–cell adhesion by promoting internalization of E-cadherin, upregulation of Rab11 signaling, and a novel PAK1-dependent phosphorylation of RabGDIβ that increases its binding to Rab5 and Rab11.

Signaling by PAK1 and Rac1 are important for the maintenance of epithelial junctions and disruption of keratinocyte adhesion (Lozano et al., 2008; Nola et al., 2011). These seemingly contradictory results are interpreted as a process that is necessary for junction homeostasis (i.e., transient PAK1 activation; Nola et al., 2011) but is derailed by abnormal Rac1 activation. Understanding these diverse Rac1 roles will help to define specific signaling that can be targeted to modulate cell–cell adhesion in a positive or negative manner.

**Figure 10.**
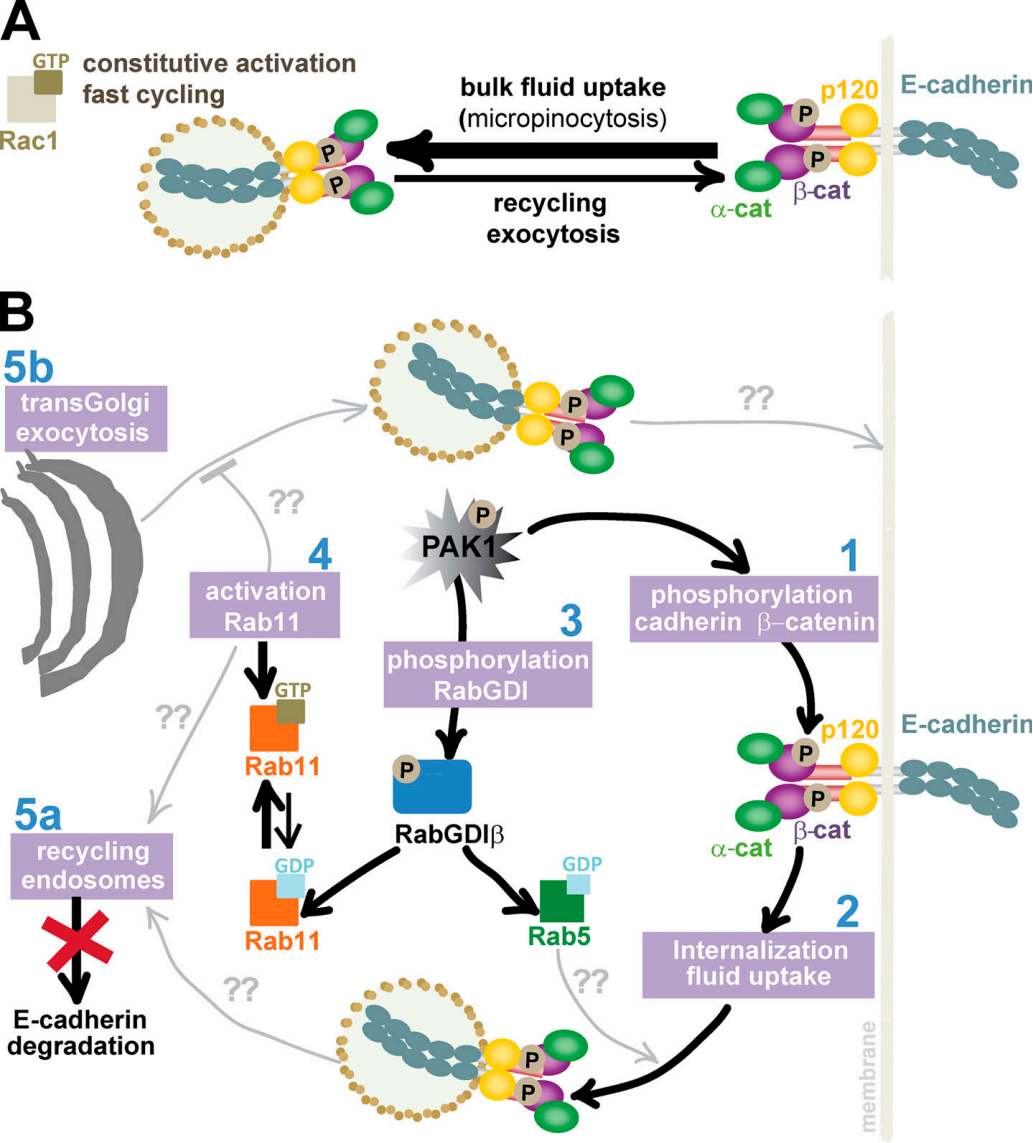
**Rac1 and PAK1 signaling imbalances internalization and exocytic routes to perturb E-cadherin levels at cell–cell contacts.** **(A)** Constitutively active Rac1 (Q61L) or fast-cycling Rac1 (P29S) reduces the surface level of E-cadherin receptors and disrupt its localization at cell–cell contacts. Active Rac1 alters the balance between the uptake of bulk membrane and the redelivery of E-cadherin to junctions and surface. **(B)** Various pathways contribute to junction perturbation. Black lines are steps demonstrated in this work, and gray lines are processes predicted to occur based on previous knowledge. The serine/threonine kinase PAK1 is activated by Rac1. Step 1: Direct phosphorylation of E-cadherin or β-catenin promotes a stronger interaction between these proteins. Step 2: Cadherin receptors are co-internalized with the catenins, most likely via bulk fluid uptake. Step 3: PAK1 directly phosphorylates RabGDIβ, which increases its interaction with a subset of inactive Rabs (i.e., Rab5 and Rab11). Step 4: Rab11.GTP levels are higher following Rac1 activation via an unknown mechanism. Step 5: The elevated activity of Rab11 and its increased retrieval from membranes by phosphorylated RabGDIβ may participate in two events—promoting recycling, thereby avoiding cadherin degradation (step 5a), and/or delaying cadherin exocytosis (step 5b).

Rac1 activation promotes the internalization of E-cadherin in a PAK1-dependent manner (this work; Fig. 10) and by a distinct mechanism compared with the classical clathrin-mediated E-cadherin turnover (Kowalczyk and Nanes, 2012; West and Harris, 2016). First, there is no dissociation of catenins from cadherin tail during internalization (this work; Akhtar and Hotchin, 2001). PAK1 phosphorylation of β-catenin (Zhu et al., 2012) and E-cadherin tail, a novel PAK1 substrate identified herein, results in a tighter association of E-cadherin and catenins (Fig. 10 B, step 1 and 2).

Second, in normal keratinocytes, E-cadherin–containing vesicles appear to be micropinosomes (around 100-nm vesicles; Fig. 10 A; Egami et al., 2014) compared with macropinosomes (large vacuoles 0.2–5 µm; Ha et al., 2016) during cadherin uptake in transformed cell lines (head and neck tumor cell lines; Akhtar and Hotchin, 2001; Akhtar et al., 2000). Macropinosomes have also been shown following EGF stimulation (Bryant et al., 2007) or the uptake of cadherin molecules not engaged in adhesion in MCF7 cells (Paterson et al., 2003; Sabatini et al., 2011). We surmise that Rac1-dependent acceleration of micropinocytosis contributes to the removal of bulk membrane, including E-cadherin receptors, and that bulk fluid uptake regulates E-cadherin surface levels (Fig. 10 A).

Micropinocytosis has no specific molecular marker, and it is thought to occur continuously to drive fluid uptake and membrane turnover, rather than internalization and degradation of specific receptors. These results entail that E-cadherin internalized via micropinocytosis may likely be diverted from degradative compartments (Bryant et al., 2007; Schill and Anderson, 2009). Alternatively, the co-internalization of cadherin in complex with catenins may mask motifs for E-cadherin breakdown during Rac1 activation as shown herein. It is, however, unclear how a nonspecific process, such as micropinocytosis, can account for the unequivocal E-cadherin adhesion destabilization. It is feasible that the balance between the higher rate of Rac1-dependent membrane internalization (i.e., bulk fluid uptake) and the specific delivery of E-cadherin (from recycling or exocytosis of biosynthetic pools) may result in reduced cadherin surface levels (Fig. 10 A; see below; Kowalczyk and Nanes, 2012; Wirtz-Peitz and Zallen, 2009).

A direct regulation of intracellular trafficking by PAK1 has not yet been explored—for example, phosphorylation of Rabs or their upstream regulators Rab (Shinde and Maddika, 2018; Steger et al., 2016). We find that PAK1 phosphorylates RabGDIβ (Fig. 10, step 3), but not the highly homologous, brain-specific RabGDIα. RabGDI facilitates continuous Rab cycling (activation) and progression of vesicular transport (Goody et al., 2005). Fusion between two intracellular compartments occurs by localized inactivation of Rabs by Tre-2/Bub2/Cdc16 (TBC) domain-containing Rab-specific GTPase Activating Proteins (TBC/RabGAPs; Frasa et al., 2012), followed by a RabGDI-dependent retrieval of inactive Rabs from fused membranes (Alory and Balch, 2001).

No specific kinase has been shown to phosphorylate RabGDIβ. The ability of PAK1 to phosphorylate both RabGDIβ (this work) and RhoGDI (Ard et al., 2012; DerMardirossian et al., 2004) strongly indicates a more general mechanism to fine-tune Rab and Rho GTPase activity with cell signaling and metabolism. RhoGDI phosphorylation by various kinases is known to reduce its affinity to specific Rho GTPases, thereby releasing the GTPase for activation (Ard et al., 2012; DerMardirossian et al., 2006; DerMardirossian et al., 2004; Dovas et al., 2010; Wu et al., 2009). Phosphorylation of RabGDI is less understood. RabGDIα phosphorylation by serum- and glucocorticoid-inducible kinase and p38 increases its binding to Rab4 or Rab5, respectively (Cavalli et al., 2001; Liu et al., 2010). Thus, in contrast to RhoGDI, RabGDI phosphorylation is associated with a stronger interaction with Rabs and modulation of specific Rab routes by promoting Rab retrieval, cycling, and relocalization (Schalk et al., 1996; Wu et al., 1998, 2009; Luan et al., 2000; Cavalli et al., 2001; Chen et al., 2009; Liu et al., 2010; Shisheva A. et al., 1999).

We find that PAK1 phosphorylates a unique RabGDIβ residue (Ser382) that maps to the Rab docking platform in the 3D structure (Cavalli et al., 2001; Liu et al., 2010; Wu et al., 1998; Wu et al., 2009; Schalk et al., 1996; Luan et al., 2000; Shisheva A. et al., 1999), suggesting a potential regulation of Rab binding. Indeed, we observe higher levels of interaction between phosphomimetic RabGDIβ with Rab5 or Rab11 (Fig. 10 B, step 3). Rab5 participates in macropinosome formation (Egami et al., 2014) and it could potentially exert the same role during micropinocytosis. The increased binding of Rab11 to phosphorylated RabGDIβ parallels the Rac1-dependent activation of Rab11 (Fig. 10 B, step 4), thereby supporting the view that Rab11 function is relevant. We surmise that Rac1 expression may imbalance E-cadherin intracellular trafficking and modulate Rab5 and Rab11 availability for cycling and activation.

At steady state, interfering with Rab11 function, per se, does not perturb E-cadherin stability in keratinocytes, similar to other Rac1 effectors, such as PAK1 or Armus (Frasa et al., 2010; Lozano et al., 2008; Nola et al., 2011). However, in the context of Rac1 expression, Rab11 activation is necessary for disruption of cell–cell contacts, as preventing endogenous Rab11 activation partially rescues junction levels. Rab11 activation may impact various trafficking pathways essential for junction stability (de Beco et al., 2009; Woichansky et al., 2016): Recycling endosomes (slow recycling) or exocytosis, including post–trans-Golgi delivery of biosynthetic proteins (Polgar and Fogelgren, 2018).

In contrast to other GTPase disruption of keratinocyte junctions (Braga et al., 2000; Brezovjakova et al., 2019; Frasa et al., 2010), Rac1 activation induces a striking phenotype: Cells flatten out with elongation of the contacting interface between neighbors (this work), and thickening of cadherin staining in the middle of junctions (Lozano et al., 2008). The progressive disappearance of cadherin staining from cell corners suggests that E-cadherin delivery to junctions is misdirected to the center of contacts. We surmise that, during Rac1-dependent contact disruption, a predicted increase in E-cadherin delivery to basolateral domains by Rab11 may not be able to overcome the bulk internalization of membrane, resulting in a net reduction of surface E-cadherin levels (Fig. 10 A).

Alternatively, epithelial trafficking regulation by Rac1 and Rab11 may be more complex than anticipated, with the distinct targeting to basolateral versus apical domains and variable phenotypes in different cell types (this work; Desclozeaux et al., 2008; Welz et al., 2014; Yan et al., 2016). Exocytic transport from the trans-Golgi network and recycling from endocytic pools may occur independently and be regulated differently by Rab11 and Rac1 (Chen et al., 1998; Lock and Stow, 2005; Woichansky et al., 2016). There is also evidence that active Rac1 expression in Madin-Darby Canine Kidney (MDCK) cells slows apically directed biosynthetic or postendocytic traffic of E-cadherin (Jou et al., 2000). Similarly, Rab11 activation on its own reduces E-cadherin surface levels in Hela and MDCK cells (Lock and Stow, 2005).

Finally, another contributor to the Rac1 phenotype on junctions could be the mistargeting of Rab11-containing vesicles to the basolateral membrane. The latter could result via two nonexclusive mechanisms: First, the participation of Rac1 and polarity complexes in the appropriate docking of endosomes to adherens junctions (Winter et al., 2012). Second, Rab11 modulation of vesicle docking to the cell membrane, either during the fusion of exocytic vesicles (via its association with the exocyst complex, Sec15) or closing pores (via its interaction with Munc13-4, SNARE complex; Polgar and Fogelgren, 2018; Takahashi et al., 2012). Perturbation of Rab11 or exocyst complex function leads to accumulation of vesicles underneath plasma membrane, unable to target their cargo. Clearly, further studies are necessary to dissect the precise mechanisms that rely on Rab11 function downstream of inappropriate Rac1 activation.

The key role of PAK1 to rewire and integrate Rab and Rho GTPase signaling via phosphorylation of their respective GDIs reinforce the importance of cooperation across the distinct classes of GTPases beyond their regulation by classical GEFs or GAPs. We unravel new principles of the integration of Rac1 and Rab GTPases to fine-tune the binding affinity of RabGDIβ, promote Rab11 activation, and alter the balance between bulk membrane turnover and E-cadherin deliver to junctions (recycling or exocytosis). The newly described role of PAK1 in intracellular trafficking is highly significant for known PAK1 functions, such as cell motility, membrane remodeling, and tumor metastasis. Furthermore, the closer interplay between cadherins, PAK1, and vesicular transport strengthens the participation and impact of Rabs (Qin et al., 2017; Banworth and Li, 2018) and RabGDI (Ming et al., 2014) on epithelial malignancies.

## Materials and methods

### Cells

Normal human keratinocytes isolated from neonatal foreskin (strain Sf, passages 3–6) were cultured as described previously (Braga et al., 1997). For RNAi experiments, keratinocytes were grown to 40–50% confluence, after which they were transfected with Rab11a siRNA [UGUCAGACAGACGCGAAAA(dT)(dT)] and Rab11b [UUUUCGCGUCUGUCUGACA(dT)(dT)] and repeated 24 h afterward. Transfection was performed in standard medium with cells at 40% confluence and expressed overnight. For biotinylation assays, normal keratinocytes were seeded on 24-well plates and grown until 70% confluence. For immunoprecipitations and pull-downs, cells were grown to 70% confluence.

Approximately 50–100 cells/coverslip were microinjected as described before (Braga et al., 1997). DNA concentrations were titrated to obtain optimal expression levels of each construct (0.2 mg/ml for Rac1). Injected constructs were expressed for 6 h. Keratinocytes were transfected using Jetprime reagent (Polyplus) at a 2 µl/µg ratio according to manufacturer’s instructions with 0.5 µg pRK5flag-Rac1^Q61L^, pEGFP-Rac^Q61L^, pEGFP-Rac^P29S^, pRFP-Rac^Q61L^, or 0.5 μg pEGFP overnight. RabGDI constructs, pEGFP-RabGDIβ^WT^, pEGFP-RabGDIβ^S382A^, and pEGFP-RabGDIβ^S382D^, were transfected using 1 µg DNA. For siRNA transfections, 20 µM siRNA of Rab11 (targeting Rab11a and Rab11b) or nontargeting control siRNA were incubated with Interferin (Polyplus) and then added to cells. Cells were treated with siRNA again after 24 h, at which point they were transfected with pEGFP-Rac^Q61L^ or pEGFP-Rac^P29S^ using Jetprime for the cotransfections (siRNA oligos and plasmids).

### Constructs and mutagenesis

The following constructs were used in this paper: pGEX-2T–α-catenin, pGEX-2T–β-catenin, pGEX–E-cadherin cytoplasmic tail (gift from Y. Fujita, Kyoto University, Kyoto, Japan), pGEX-PAK kinase domain (gift from M. Nikolic), pTAT-HA-Rac1^Q61L^ and pTAT-HA-Rac1^T17N^ (from S. Dowdy, University of California, San Diego, La Jolla, CA), and pGEX-Rab11-FIP3 (gift from E. Hirsch, University of Torino, Torino, Italy; Franco et al., 2014). Mammalian expression vectors were: pRK5-flag-Rac1^Q61L^, pEGFP-Rac^Q61L^, pRFP-Rac^Q61L^, pRK5myc-PAK^AID^, pEGFP-Rab11^S20V^ (constitutively active), and pEGFP-Rab11^S25N^ (dominant negative); and pcDNA-myc-β-catenin (gift from Y. Fujita).

The AID of PAK1 was subcloned from pRK5myc-PAK^AID^ (aa 83–149) into the pTAT-HA vector. Site-directed mutagenesis (Stratagene’s QuickChangeII site-directed mutagenesis kit) was performed according to the manufacturer’s conditions to obtain the point mutants T551A, S552A, and S675A in pGEX-2T–β-catenin, and the mutations S675A and S675D in pcDNA-myc–β-catenin. Mutations S382D and S382A were introduced in WT pGEX-RabGDIβ to generate phosphomimetic and nonphosphorylatable versions. Mutagenesis of RabGDIβ was performed to obtain RabGDIβ^S382A^ and RabGDIb^S382D^, which were then subcloned into pEGFP for use in transfections. The fast-cycling oncogenic Rac^P29S^ was subcloned into pEGFP vector from pRetroX-Rac^P29S^ (gift from A. Malliri,  Cancer Research UK Manchester Institute, Manchester, UK; Krauthammer et al., 2012).

### Antibodies, immunostaining, and immunoprecipitation

E-cadherin staining was performed using ECCD-2 (rat monoclonal; Zymed) or HECD1 (mouse monoclonal) antibody. Additional mouse monoclonal antibodies used were anti-flag (M2; Sigma-Aldrich), anti-myc (9E10; Upstate), anti-actin (C4; MP Biomedicals), anti–β-catenin (Life Technologies), anti-Rab11 (recognizes Rab11a and Rab11b; BD Transduction Laboratories), anti-caveolin 1 (2297; BD Transduction Laboratories), anti-CD63, and anti-EEA1 (BD Transduction Laboratories). Rabbit polyclonal antibodies used were against α-catenin (VB1), β-catenin (VB2), β1-integrin (cytoplasmic tail; provided by F. Giancotti), phospho–β-catenin (S675; D2F1; Cell Signaling Technology), Rab5 (Abcam), Rab7 (Cell Signaling Technology), Rab22 (Abcam), GFP (Abcam), and RabGDIα and RabGDIβ (Santa Cruz Biotechnology). Cells were incubated with Transferin-Alexa Fluor 568 (Invitrogen), Texas Red-BSA (Invitrogen), or Texas Red-Dextran (Invitrogen). Secondary antibodies were bought from Jackson ImmunoResearch Laboratories (Stratech Scientific) or Pierce.

For characterization of E-cadherin endocytosis pathway, cells microinjected with Rac1 cDNA plasmid and after 5.5 h of expression incubated for 30 min with Transferrin-Alexa Fluor 568 (100 µg/ml), Texas Red-BSA (50 µg/ml), or Texas Red-Dextran (100 µg/ml).

Immunostaining was performed as described in Braga et al. (1997). Images were collected on a Leica SP5 upright confocal microscope using Leica LAS AF Lite software or Zeiss LSM 510 inverted confocal. To avoid leakage between different filters, the laser was optimized for each fluorophore and images collected separately. Alternatively, images were collected using a widefield Olympus PROVIS BX51 microscope using a 60×/1.40 Oil PlanApo ∞/0,17 (lens from Olympus) and SimplePCI 6 software (Hamamatsu).

For immunoprecipitation, keratinocytes were washed in cold PBS and lysed in 500 µl lysis buffer (1% Triton X-100, 20 mM Hepes [pH 7.4], 500 mM NaCl, 10 mM NaF, 1 mM Na-pyruvate, 1 mM Na-orthovanadate, 4 mM b-glycerol-phosphate and protease inhibitors). After centrifugation of lysates at 14,000 rpm at 4°C for 5 min, samples were precleared with protein G or protein A slur. Anti–E-cadherin antibodies were bound to Protein G or anti–β-catenin antibodies to protein A and incubated with precleared supernatants for 45 min at 4°C. Beads were washed three times in lysis buffer, loaded onto SDS-PAGE, and analyzed by Western blot.

### Biotinylation assay

TAT-fusion proteins were added to the cells with 2-d-old standard medium and incubated at 37°C for 4 h (40 µg TAT-Rac^Q61L^; 60 µg TAT-PAK^AID^ (aa 83–149); 40 µg TAT-Rac^T17N^; 100 µg TAT). For biotinylation assay, cells were treated with TAT (YGRKKRRQRR R, synthesized by CRUK) and TAT-fusion proteins before (surface protein measurements) or after cell surface biotinylation (internalized protein measurements). For detection of internalized protein, cells were washed with PBS containing 10 mM CaCl_2_ and 1 mM MgCl_2_ and incubated with 0.5 mg/ml EZ-Link NHS-SS-Biotin (Pierce) for 30 min on ice, followed by washing with quenching reagent (15 mM glycine in PBS-Ca-Mg). After biotinylation, cells were incubated at 37°C with 2-d-old standard medium (previously removed from the cells) containing TAT-proteins for various time points to allow for endocytosis. Biotinylated proteins on the plasma membrane were then stripped at 0°C by glutathione treatment twice for 15 min (60 mM glutathione, 75 mM NaCl, 10 mM EDTA, 75 mM NaOH, and 1% BSA). Cells were lysed in radioimmunoprecipitation assay buffer (150 mM NaCl, 20 mM Tris 7.4, 0.1% SDS, 1% Triton, 0.5% deoxycholate, 5 mM EDTA) with protease inhibitors, and an aliquot was separated to measure the total amount of E-cadherin. Internalized biotinylated proteins were recovered from lysates by coprecipitation with streptavidin beads. The amount of internalized and total E-cadherin was quantified by Western blot.

To determine surface protein levels, keratinocytes were first treated with TAT-proteins for various time points, and then surface biotinylation was performed, followed by washing with quenching reagent and PBS. Cells were lysed in radioimmunoprecipitation assay buffer, and surface biotinylated proteins were recovered using streptavidin beads and identified by Western blot. Quantification of the total level of E-cadherin was normalized to actin expression. Values of total E-cadherin and surface proteins at time x (t_X_ − level observed in time x) were quantified as a ratio of the amount at time 0 (t_0_). Internalized proteins were quantified as a ratio of the amount at time x (t_X_) to the amount of internalized protein after 2 h (t_2_).

### Production and purification of recombinant fusion proteins

For GST-fusion proteins, bacteria were cultured overnight and induced with 0.3 mM IPTG (Calbiochem) for 3–5 h at 30°C or overnight at 16°C. The bacterial pellet was resuspended in lysis buffer (50 mM Tris HCl [pH 7.5], 100 mM NaCl, 5 mM DTT, 1 mM PMSF; a cocktail of protease inhibitors leupeptin, pepstatin, and pefabloc at 5 µg/ml each). Proteins were dialyzed against dialysis buffer (15 mM Tris HCl [pH 7.5], 150 mM NaCl, and 0.1 mM DTT). His-tagged TAT-fusion proteins were purified from bacteria pellet lysed in Lysis Buffer (standard buffer [SB] 3 mM MgCl_2_, 5 µg/ml lysozyme, 10 µg/ml DNase, 1 mM PMSF; cocktail of protease inhibitors, as above) on Ni-charged chelating-sepharose beads (GE Healthcare) followed by washes with SB (30 mM Tris HCl [pH 7.5], 100 mM NaCl, 1 mM β-mercaptoethanol, 5 mM MgCl_2_) plus 20 mM imidazole. His-tagged fusion proteins were eluted from beads with elution buffer (SB + 1 M imidazole) and dialyzed against SB. GST-FIP3 (Franco et al., 2014) and GST-p120^CTN^ were prepared as described.

### In vitro kinase assay

Recombinant PAK1 kinase domain (4 µg) was incubated with 10 µCi [γ-^32^P]-ATP (PerkinElmer) and GST-fusion proteins (0.4 nmol) trapped on glutathione-sepharose beads in phosphorylation buffer (50 mM Hepes [pH 7.3], 10 mM MgCl_2_, 10 mM sodium fluoride, and 2 mM MnCl_2_) containing 40 µM cold ATP. Reaction was incubated for 5 min at 30°C and terminated by washes with phosphorylation buffer and addition of SDS sample buffer. Proteins were separated in SDS-PAGE gel and phosphorylation was visualized by autoradiography. Alternatively, kinase assay was also used for RabGDIβ-phosphorylation detection with nonradioactive ATP and samples run on Phos-tag gels (Wako Pure Chemical Industries) to determine whether phosphorylation had occurred. For identification of phosphorylation sites, in vitro kinase reactions were analyzed by mass spectrometry (commercially, FingerPrints Proteomics, Dundee University).

### In vitro binding assay

For GST pull-down assay, recombinant GST-fusion protein (catenins or cadherin cytoplasmic tail) was coupled for 1 h at 4°C to 20 µl glutathione-Sepharose beads (GE Healthcare), and then beads were washed with phosphorylation buffer (50 mM Hepes [pH 7.3], 10 mM MgCl_2_, 10 mM sodium fluoride, and 2 mM MnCl_2_) and incubated with 4 µg recombinant PAK1 kinase domain and 10 mM cold ATP for 5 min at 30°C. Reaction was terminated by washes with ice-cold phosphorylation buffer. After kinase reaction, beads were incubated with different concentrations of cleaved proteins (0.05 µg and 0.1 µg in phosphorylation buffer containing 0.3 M NaCl and 0.5% Triton X-100) for 30 min at 4°C, followed by washing three times with phosphorylation buffer plus 0.3 M NaCl and 0.5% Triton X-100. Proteins bound to beads were eluted with SDS sample buffer containing freshly added DTT (0.1 M) and analyzed by SDS-PAGE and immunoblotting. Alternatively, protein complexes were formed in vitro before PAK1 phosphorylation.

GST-RabGDIβ^WT^, GST-RabGDIβ^S302A^, and GST-RabGDIβ^S302D^ were used to pull down endogenous Rabs from keratinocyte lysates. Briefly, cells were lysed in lysis buffer (50 mM Tris HCL, 0.5% NP-40, 150 mM NaCl, 1 mM MgCl_2_, 1 mM EDTA, 1 mM PMSF, and protease inhibitor cocktail, as above). Lysates were incubated with GST-RabGDIβ beads for 1 h at 4°C and washed, and samples were used for Western blots to detect associated Rabs.

To determine levels of Rab11 activation, cells were transfected with constitutively active Rac1 constructs overnight, washed in cold PBS, and lysed in 50 mM Tris HCL (pH 7.4), 100 mM NaCl, 1% NP-40, 10% glycerol, 10 mM MgCl_2_, 10 mM sodium fluoride, 1 mM sodium orthovanadate, 1 mM PMSF, and protease inhibitor cocktail, as above. Samples were processed for pull downs using GST-FIP3 essentially as described in Franco et al. (2014), except for shorter spins of 2 min and three washes after the pull-down.

### Quantification and statistics

Quantification of junction phenotypes used a semiautomated custom-made software, Junction Mapper (Brezovjakova et al., 2019). Quantification of the levels of E-cadherin at junctions was performed using the parameter Coverage Index that measures the proportion of the length of contacting interface that is covered by cadherin staining (Lozano et al., 2008), the percentage of the area of the contacting interface that contains pixels of the junction marker, measurement of the pixel length of contacting interface, and length of the staining of the junction marker. Images were processed using Adobe Photoshop and Adobe Illustrator. Colocalization parameters were measured using SimplePCI 6 software.

Western blot films in the linear range exposure were scanned and identified bands quantified using WCIF ImageJ software. Quantification of the levels of active Rab11 were performed as described (Nola et al., 2011). Levels of endogenous Rab11 associated with GST-FIP3 (active Rab11) were then normalized to the total levels of Rab11 found in the respective lysates. Samples expressing GFP were used to calculate the basal line of Rab11 activation and arbitrarily set as one. Stimulated samples were expressed as fold change to levels found in GFP samples (basal levels). RabGDIβ interaction with distinct Rabs was quantified in a similar way by normalizing the levels of associated Rabs with mutants S382A or S382D to the amount detected associated with WT RabGDIβ and expressed as fold change.

Error bars represent error of the means, unless stated otherwise in figure legends. Statistical analysis was performed with *t* tests or Kruskal-Wallis with Dunn's multiple comparison test using GraphPad Prism. Data distribution was assumed to be nonparametric, but this was not formally tested.

### Online supplemental material

Fig. S1 shows cellular effects of TAT proteins used in this study. Fig. S2 shows that PAK1 phosphorylation of β-catenin does not participate in junction perturbation in keratinocytes. Fig. S3 shows the search for putative phosphorylation sites in RabGDIβ. Fig. S4 shows the alignment of RabGDIα and RabGDIβ from human, bovine, and mouse species. Fig. S5 shows that localization of RabGDIβ or Rab11 mutants at junctions does not impair cell–cell contacts.
